# A divergent protein kinase A regulatory subunit essential for morphogenesis of the human pathogen *Leishmania*

**DOI:** 10.1371/journal.ppat.1012073

**Published:** 2024-03-29

**Authors:** Renana Fischer Weinberger, Sabine Bachmaier, Veronica Ober, George B. Githure, Ramu Dandugudumula, Isabelle Q. Phan, Michal Almoznino, Eleni Polatoglou, Polina Tsigankov, Roni Nitzan Koren, Peter J. Myler, Michael Boshart, Dan Zilberstein

**Affiliations:** 1 Faculty of Biology, Technion - Israel Institute of Technology, Haifa, Israel; 2 Faculty of Biology, Genetics, Ludwig-Maximilians Universität München, Martinsried, Germany; 3 Seattle Structural Genomics Center for Infectious Disease, Seattle, Washington, United States of America; 4 Center for Global Infectious Disease Research, Seattle Children’s Research Institute, Seattle, Washington, United States of America; 5 Department of Pediatrics, Department of Biomedical Informatics & Medical Education, and Department of Global Health, University of Washington, Seattle, Washington, United States of America; San Diego State University, UNITED STATES

## Abstract

Parasitic protozoa of the genus *Leishmania* cycle between the phagolysosome of mammalian macrophages, where they reside as rounded intracellular amastigotes, and the midgut of female sand flies, which they colonize as elongated extracellular promastigotes. Previous studies indicated that protein kinase A (PKA) plays an important role in the initial steps of promastigote differentiation into amastigotes. Here, we describe a novel regulatory subunit of PKA (which we have named PKAR3) that is unique to *Leishmania* and most (but not all) other Kinetoplastidae. PKAR3 is localized to subpellicular microtubules (SPMT) in the cell cortex, where it recruits a specific catalytic subunit (PKAC3). Promastigotes of *pkar3* or *pkac3* null mutants lose their elongated shape and become rounded but remain flagellated. Truncation of an N-terminal formin homology (FH)-like domain of PKAR3 results in its detachment from the SPMT, also leading to rounded promastigotes. Thus, the tethering of PKAC3 *via* PKAR3 at the cell cortex is essential for maintenance of the elongated shape of promastigotes. This role of PKAR3 is reminiscent of PKARIβ and PKARIIβ binding to microtubules of mammalian neurons, which is essential for the elongation of dendrites and axons, respectively. Interestingly, PKAR3 binds nucleoside analogs, but not cAMP, with a high affinity similar to the PKAR1 isoform of *Trypanosoma*. We propose that these early-diverged protists have re-purposed PKA for a novel signaling pathway that spatiotemporally controls microtubule remodeling and cell shape.

## Author summary

*Leishmania* are single cell intracellular parasites of mammals, including humans. During their life, they cycle between macrophage phagolysosomes where they reside as rounded amastigotes, and the midgut of female sand flies, which they colonize as elongated promastigotes. Adaptation of *Leishmania* parasites to life in their mammalian host includes differentiation-associated changes in morphology from elongated promastigotes to rounded amastigotes. This study shows that a novel trypanosomatid-specific protein kinase A regulatory subunit (PKAR3) is essential for maintenance of the elongated shape of *Leishmania* promastigotes. PKAR3 is anchored to the subpellicular microtubule in the cell cortex by an N-terminal formin homology (FH)-like domain; where it recruits a developmentally regulated catalytic subunit (PKAC3). Exposure to the differentiation signal results in a rapid downregulation of PKA activity and subsequently cell rounding. Thus, the cAMP-independent PKAR3 appears to have been repurposed to serve an AKAP-like role in spatiotemporal control of morphogenesis, highlighting the evolutionary diversity of this family of signalling proteins.

## Introduction

*Leishmania donovani* is a (trypanosomatid) protozoan parasite that causes kala azar, a fatal form of visceral leishmaniasis in humans [1]. These parasites cycle between the midgut of female sand flies, where they reside as flagellated elongated extracellular promastigotes, and the phagolysosomes of mammalian macrophages, where they live as rounded amastigotes that lack a visible flagellum [2]. Infective (metacyclic) promastigotes are introduced into the host during the sand fly blood meal after which they are phagocytosed by resident macrophages near the bite site [3,4]. Once inside the host phagolysosome, promastigotes encounter two physical cues that distinguish this environment from that of the vector gut: acidic pH (~5.5) and elevated temperature (~37°C). Promastigotes process these cues into a signal that initiates differentiation into amastigotes [5–7].

Among the earliest events during *L*. *donovani* promastigote-to-amastigote differentiation are changes in the phosphorylation profile of many proteins [8]. Significantly, while protein kinase A (PKA)-specific phosphorylation is common in promastigotes, most of these phosphoproteins are dephosphorylated within a few minutes after initiation of differentiation into amastigotes [7]. This suggests that regulation of PKA activity is important for regulation of *Leishmania* differentiation. PKA-mediated signaling pathways are ubiquitous in eukaryotic cells, being implicated in growth control, development, and metabolism. The canonical cAMP dependent PKA regulatory system in vertebrates involves assembly of catalytic (PKAC) and regulatory (PKAR) subunits into an inactive heterotetrametric complex. This interaction is mediated by a dimerization and docking (D/D) domain in the N-terminal portion of PKAR [9]. When cAMP binds the cyclic nucleotide-binding domains (cNBDs) near the C-terminal of each PKAR, the resultant conformational change triggers the dissociation of the PKAC/R complex and the release of catalytically active PKAC subunits. Compartmentation of PKA activity to different cellular microdomains is achieved (at least in higher eukaryotes) through interaction of the D/D domain of PKAR with a diverse family of A-kinase anchoring proteins (AKAPs) that vary according to biological context [10]. However, PKARs from many lower eukaryotes (including trypanosomatids) appear to lack a canonical D/D domain [11,12] and any obvious AKAPs [13].

Most eukaryote genomes (with the notable exception of plants and algae) encode one or more PKAC subunits [13]. Trypanosomatids (including *L*. *donovani*) are no exception, having two subunits (PKAC1 and C2) that are 99% identical and a third (PKAC3) that is more divergent [14–16]. Phosphoproteomic analyses of *L*. *donovani* revealed that all three PKAC subunits contain at least two phosphorylation sites [17]. One, within the kinase active site loop, matches the canonical PKA phosphorylation motif (RXXpS/pT) and is phosphorylated only in promastigotes. The second is located within a canonical ERK1/ERK2 substrate motif (pSP) close to the C-terminus and while PKAC1/C2 is phosphorylated at this site in promastigotes, PKAC3 is phosphorylated only after exposure to the differentiation signal [8]. In other eukaryotes, phosphorylation of both a threonine (T_197_) in the activation loop and a serine (S_338_) near the C-terminus is necessary for full activation of PKAC, suggesting that PKAC1/C2 are active in *L*. *donovani* promastigotes, while PKAC3 is transiently activated only upon exposure to the differentiation signal.

Most eukaryotes also encode one or more PKAR subunits, whose number and evolution are more dynamic than PKAC [13]. All trypanosomatids have a regulatory subunit (PKAR1) that has been shown to localize to the flagellum of *Trypanosoma brucei* [18–20] and *L*. *donovani*, where it is expressed only in the promastigote stage [7,21]. However, in contrast to higher eukaryotes, *T*. *brucei* PKAR1 does not dimerize and is cAMP-independent but binds to nucleoside analogues [11, 12, 61].

Interestingly, *L*. *donovani* (along with other *Leishmania* species and *Trypanosoma cruzi*, but not *T*. *brucei*) have a second PKAR-like subunit [14], containing at least 10 sites that are phosphorylated or dephosphorylated during differentiation, implying that it may have a regulatory role [8]. Here, we show that this protein (which we have renamed PKAR3) forms a holoenzyme complex with PKAC3 and anchors it to subpellicular microtubules at the cell cortex *via* a formin homology (FH2)-like domain at the N-terminus of PKAR3. Promastigotes of *L*. *donovani* null mutants lacking either PKAR3 or PKAC3 are predominantly rounded compared to elongated wild type (WT) cells, suggesting that tethering of PKA kinase activity to microtubules is necessary for the maintenance of elongated cell shape. Thus, this study ties together an evolutionary divergent PKA complex localized to the microtubule cytoskeleton of *Leishmania* with developmental morphogenesis.

## Results

### Leishmania PKA regulatory subunits are evolutionary divergent

**Fig 1:**
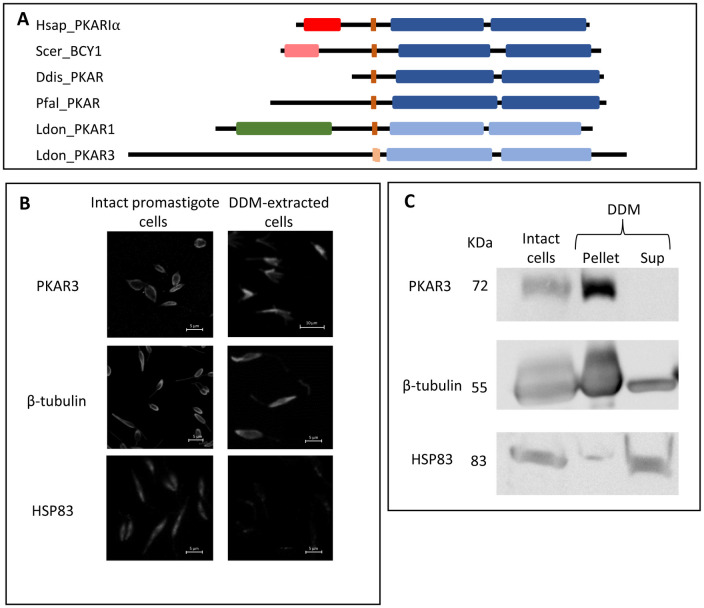
PKAR3 is anchored to the DDM-insoluble subpellicular microtubules (SPMT) at the cortex of *L*. *donovani* cells. **(A**) Schematic representation of selected *Homo sapiens* (Hsap_PKARIa), *Saccharomyces cerevisiae* (Scer_BCY1), *Dictyostelium discoideum* (Ddis_PKAR), *Plasmodium falciparum* (Pfal_PKAR) and *Leishmania donovani* (Ldon_PKAR1, Ldo_PKAR3) PKAR proteins. The scheme shows the dimerization and docking (D/D) domain (red), the RNI-like LRR domain (green), pseudo- inhibitor site (brown), and cyclic nucleotide binding (CNB) domains (blue). Orange and light blue indicate non-canonical D/D and CNB domains, respectively. (**B**) Indirect immunofluorescence using antibodies against PKAR3 (upper row), β-tubulin (middle row) and HSP83 (bottom row) in intact promastigote cells (left column) or the DDM-extracted cytoskeletons containing the SPMT (right column). Each picture is representative of at least 3 independent stainings. Note that for better visibility, the original scale bars were overpainted (**C)** Proteins from intact or DDM-extracted (supernatant and pellet) promastigotes were separated on 10% SDS-PAGE and the blotted proteins were subjected to western analysis using the antibodies indicated on the left side of the panels. The full-length gels are shown in S9 Fig.

Previous analyses of PKAR subunits from a wide range of organisms revealed that they have undergone dynamic and divergent evolution during eukaryote history [13]. We used Blast searches to identify PKAR orthologues in 15 different genera (and two unclassified trypanosomatids) from the Kinetoplastidae (S1 Table). *Leishmania* and most other trypanosomatids contain two paralogues (PKAR1 and PKAR3) that lack the canonical dimerization and docking (D/D) domain found in the N-terminal region of the PKARs of many (but not all) eukaryotes (Fig 1A). Instead, PKAR1 has a Ribonuclease Inhibitor (RNI)-like domain containing Leucine Rich Repeats (LRRs) that may mediate different protein-protein interactions but not homodimerization [11,12]. PKAR3 has a long N-terminal region without obvious domain profile signatures. Construction of a phylogenetic tree (S1 Fig) revealed that PKAR1 and PKAR3 represent two separate branches distinct from that containing the canonical PKAR(s) found in most other eukaryotes.

### Cytoskeletal localization of PKAR3 to cortical subpellicular microtubules

As shown in S2A Fig, polyclonal antibodies raised in rabbits against recombinant PKAR3 reacted with a 72 kDa protein in wild-type (WT) cells. This protein was absent in null mutants (Δ*pkar3*) but reappeared after ectopic expression of full-length *PKAR3* in the null mutant (Δ*pkar3*::*PKAR3*_*FL*_). Confocal microscopy using the antibody against PKAR3 revealed its localization to the cell cortex in *Leishmania* promastigotes (Fig 1B). The cell cortex contains a rigid cytoskeleton made of stable subpellicular microtubules (SPMT) that are composed largely of tubulin [22–24]. Previous studies have shown that non-ionic detergents can solubilize the trypanosomatid surface membrane while leaving the SPMT intact [25–27]. Treatment of *L*. *donovani* promastigotes with the zwitterionic detergent n-dodecyl β-D-maltoside (DDM, which is neutral at pH 7) resulted in enrichment of β-tubulin in the DDM-insoluble fraction (Fig 1B and 1C). In contrast, cytosolic HSP83 [28] is absent from the DDM-insoluble fraction. Likewise, the presence of PKAR3 in the DDM-insoluble pellet suggests that it is associated with the SPMT.

**Fig 2:**
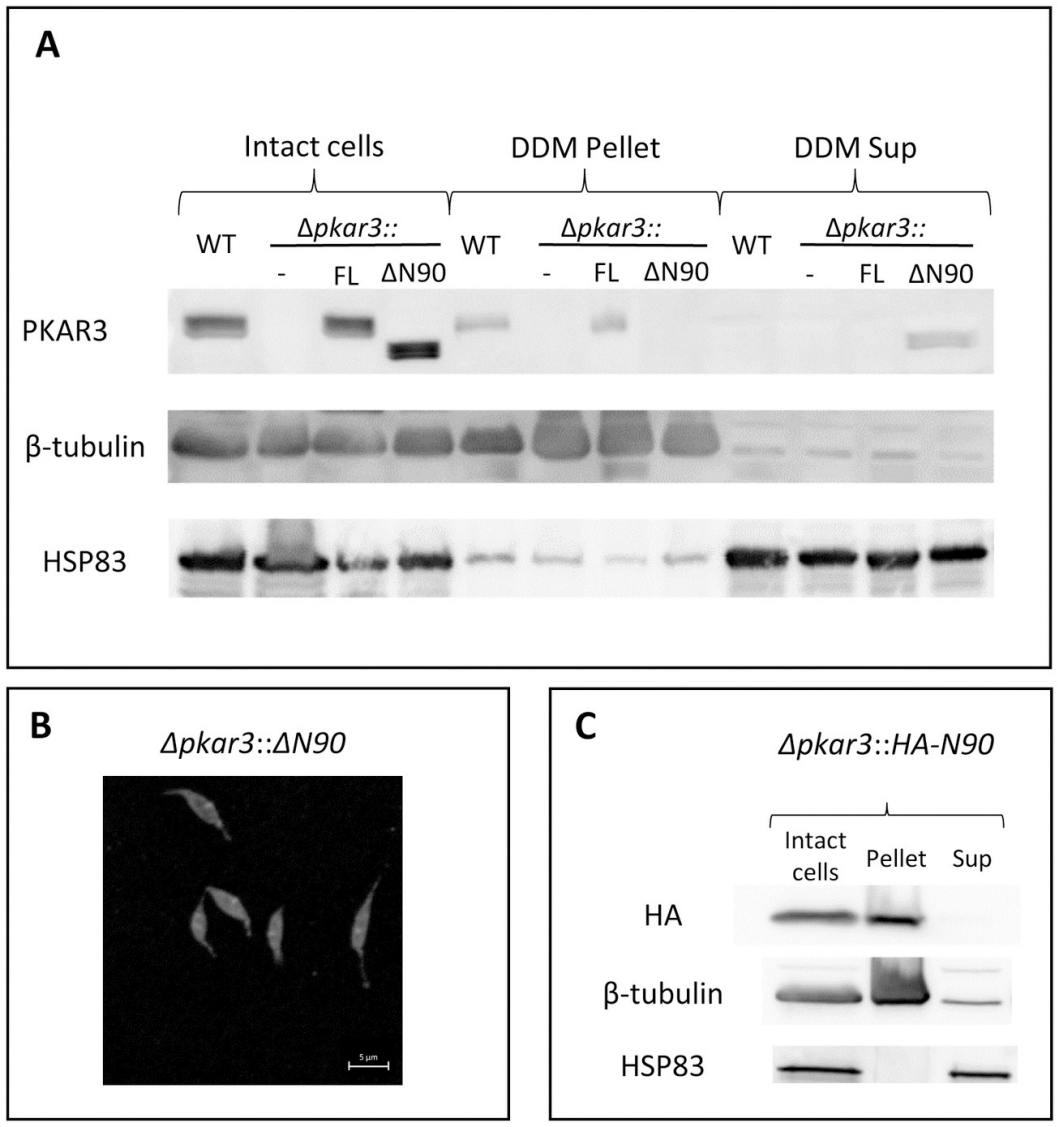
PKAR3 binds to the SPMT *via* an N-terminal putative FH2 domain. (**A**) Proteins extracted from intact and DDM-treated (supernatant and pellet) *L*. *donovani* promastigotes of the wild type (WT), *PKAR3* null mutant (*Δpkar3*), as well as *Δpkar3* ectopically expressing the full length PKAR3 (*Δpkar3*::*FL*) or the 90 aa truncated PKAR3 (*Δpkar3*::*ΔN90*). These proteins were separated by 10% SDS-PAGE and subjected to Western blot analysis using anti-PKAR3 (WT = 72 kDa, N90 = 70 kDa, upper row), anti-β-tubulin (middle row), and anti-HSP83 (lower row). The full-length gels are shown in S10A Fig. (**B**) Immunofluorescence of PKAR3 in *Δpkar3*::*ΔN90* promastigotes using antibodies against PKAR3. Each picture is representative of at least 3 independent experiments. Note that for better visibility the original scale bars were overpainted. (**C)** Proteins extracted from intact or DDM-treated promastigotes of *Δpkar3* expressing only the PKAR3 N-terminal 90 amino acids (*Δpkar3*::*HA-N90*). Proteins were analyzed by Western blotting using antibodies against the HA-tag (upper row), β-tubulin (middle row) and HSP83 (lower row). The full-length gels are shown in S10B Fig.

Binding of a PKA regulatory subunit (PKARIβ) to stable microtubules has been documented in neuronal axons of mammalian cells [29] and is mediated by interaction of the dimerization and docking (D/D) domain of PKARIβ with a microtubule-associated protein 2 (MAP2) [30]. The *Leishmania* genome does not contain an obvious orthologue of MAP2 and PKAR3 lacks the canonical D/D domain, suggesting its localization to the SPTM is mediated by a different mechanism. The N-terminal 50 amino acids of PKAR3 are relatively well-conserved and analysis using HHpred [31,32] revealed structural similarity to three Protein Data Bank (PDB) entries containing a formin homology (FH2) domain (S3 Fig). Formins are a diverse family of proteins that participate in polymerization of actin and assembly of microtubule cytoskeletons in many eukaryotes [33], leading us to speculate that this domain might be responsible for the binding of PKAR3 to the subpellicular microtubules. To test this hypothesis, we generated null mutants of *L*. *donovani PKAR3* ectopically expressing a version of *PKAR3* lacking the N-terminal 90 amino acids (*Δpkar3*::*PKAR3*_ΔN90_). Western blot analysis confirmed that both the full-length and truncated proteins are present in whole cell lysates (Fig 2A). However, only full-length PKAR3 remains bound to the DDM-insoluble pellet extracted from these cells, and the truncated protein was present only in the DDM supernatant, suggesting that it no longer binds the SPMT. Fluorescence microscopy using antibodies against PKAR3 confirmed that the truncated protein no longer localized to the cortex, being spread throughout the promastigote cell body (Fig 2B). Conversely, when a plasmid containing the N-terminal 90 amino acids of PKAR3 (with a C-terminal HA-tag) was ectopically expressed in the *Δpkar3* mutant *(Δpkar3*::*PKAR3*_N90_*)*, the fusion protein was enriched in the DDM-insoluble pellet (Fig 2C). Taken together, these results indicate that the N-terminus containing the FH2-like domain is necessary and sufficient for detergent-resistant binding of PKAR3 to the microtubule cytoskeleton.

**Fig 3:**
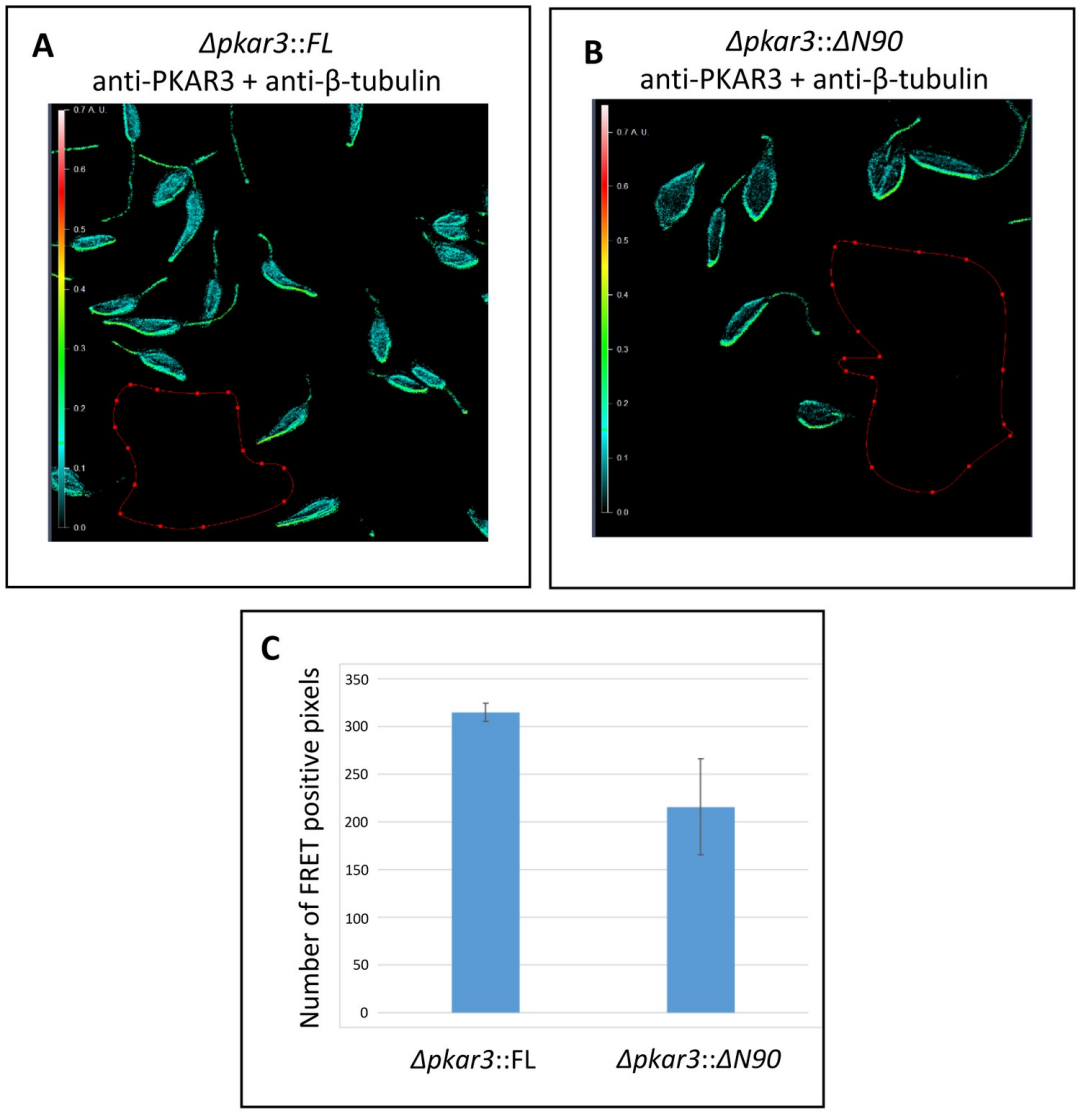
PKAR3 associates with tubulin of the SPMT. **(A)** Promastigotes of *L*. *donovani Δpkar3*::*FL* labeled with rabbit anti-PKAR3 and mouse anti-β-tubulin, followed by secondary antibody detection with goat anti-rabbit antibodies conjugated to Alexa Fluor 568 and goat anti-mouse antibodies conjugated to Alexa Fluor 647. Fluorescence Resonance Energy Transfer (FRET) emission intensity was calculated relative to the darkness within the red borders. The levels of emission intensities are color coded (see rainbow ruler on the left) according to values obtained from the Axio Vision program (ranging between 0–0.7). (**B)** FRET analysis between PKAR3 and β-tubulin in *Δpkar3* cells expressing *Δpkar3*::*ΔN90* (*Δpkar3*::*ΔN90*), carried out as in panels A. (**C**) Comparing the number of FRET positive pixels per cell between *Δpkar3*::*FL* (panel A) and *Δpkar3*::*ΔN90* cells (panel B). One-way ANOVA indicated that the number of FRET positive pixel containing *Δpkar3*::*ΔN90/*β-tubulin were 30% less than *Δpkar3*::*FL/* β-tubulin (P<0.05, n = 3).

To further characterize the association of PKAR3 with the SPMT, we used Fluorescence Resonance Energy Transfer (FRET) to determine whether PKAR3 and β-tubulin are located close together within individual promastigotes (Fig 3). The high value for corrected acceptor FRET intensity observed when PKAR3 and β-tubulin are co-expressed shows that they co-localize within the SPMT (Fig 3A). The low corrected acceptor FRET intensity in the *Δpkar3* mutant validates the approach (S4A Fig). Similarly, we saw low corrected acceptor FRET intensity between β-tubulin and an amino acid transporter [34] located in the cell membrane (S4B Fig). In contrast to the finding above that N-terminal truncated PKAR3 was not retained in the SPMT pellet after DDM treatment, there was significant corrected acceptor FRET detected in *Δpkar3*::*PKAR3*_ΔN90_ cells (Fig 3B). However, the number of pixels per cell above the threshold FRET emission intensity value and therefore qualified as “interacting” was ~30% less than obtained with PKAR3_FL_ (Fig 3C). As the concentration of ectopically expressed PKAR3_FL_ and PKAR3_ΔN90_ proteins was high in these cells (Fig 2A), other regions of PKAR3 may also interact with microtubules (albeit more weakly), especially in the absence of detergent. The FRET analyses support our hypothesis that PKAR3 is attached to the SPMT *via* direct or indirect interaction with β-tubulin.

### PKAR3 associates with PKAC3 to form a holoenzyme complex

**Fig 4:**
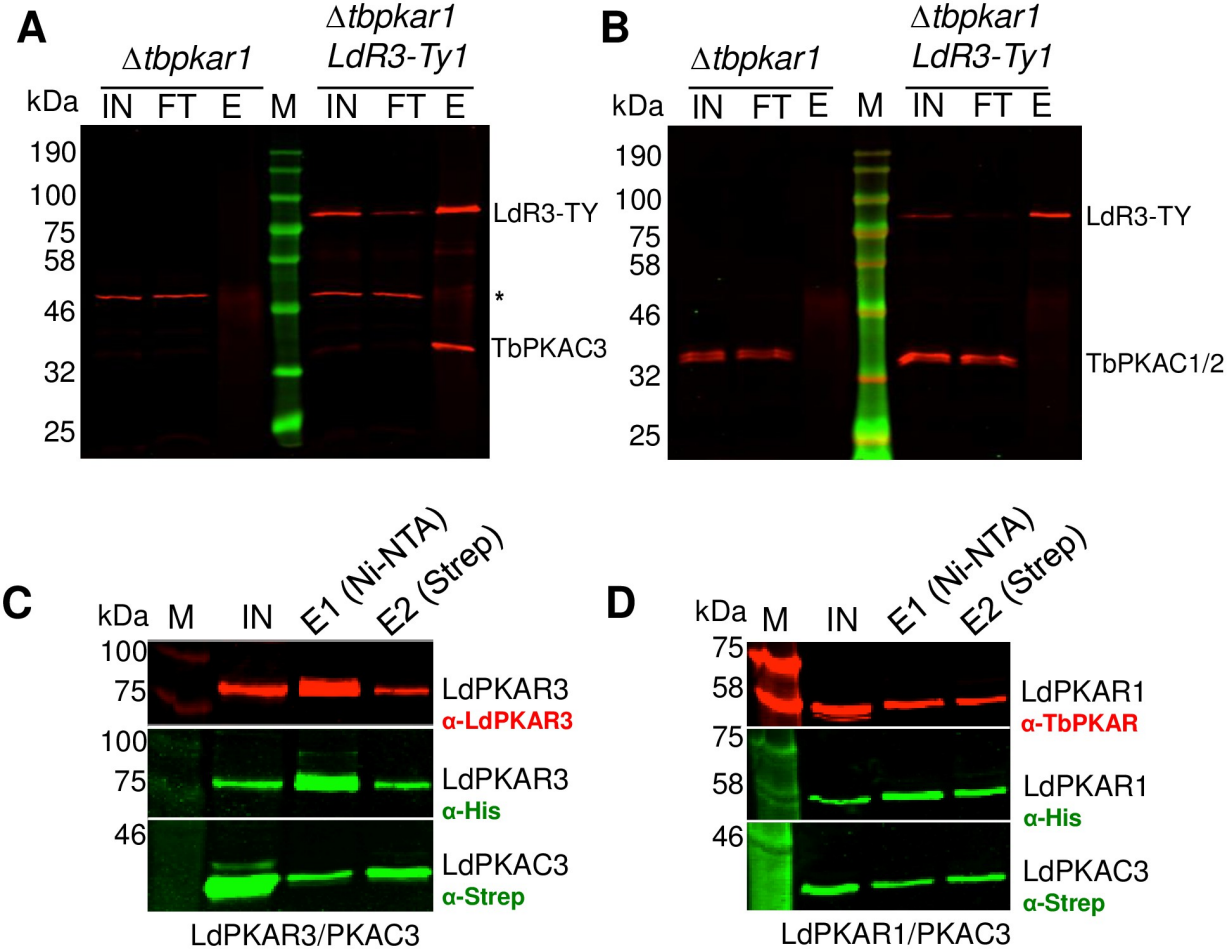
PKAR3 associates with PKAC3. (**A**) Proteins from the DDM-soluble fractions of the *T*. *brucei* MiTat 1.2 *PKAR1* null mutant (7) parental cell line (*Δtbpkar1*) and a line expressing a tetracycline-regulatable version of *L*. *donovani PKAR3* containing a C-terminal Ty1-tag (*LdR3-Ty1*) were immunoprecipitated with mouse monoclonal antibodies against Ty1, followed by western blotting with rabbit antibodies against *L*. *donovani* PKAR3 and *T*. *brucei* PKAC3. The following abbreviations denote the different fractions interrogated: IN (input), FT (flow-through), and E (elution). The molecular weight marker is indicated in kilodaltons (kDa). An unidentified protein that cross-reacts with anti-PKAC3 antibody is indicated by an asterisk. (**B**) The same samples were probed with antibodies against PKAR3 and PKAC1. (**C**) *L*. *donovani* PKAR3 and PKAC3 containing N-terminal His_6_ and Strep tags, respectively, were co-expressed in *L*. *tarentolae* and soluble proteins affinity purified on tandem Ni-NTA and Strep-Tactin columns. Aliquots from the input (IN) and eluate (E1, E2) fractions were analyzed by western blotting with rabbit antibodies against LdPKAR3, as well as mouse monoclonal antibodies against the His (Bio-Rad) and Strep (Qiagen) tags. (**D**) A similar experiment performed using tagged *L*. *donovani* PKAR1 and PKAC3 and probed with rabbit antibodies against TbPKAR1 that cross-react with LdPKAR1.

Because antibodies against *L*. *donovani* PKA catalytic subunits are not available, we initially examined whether PKAR3 could form a complex after heterologous expression in *T*. *brucei*. A Ty1-tagged version of *L*. *donovani* PKAR3 (LdPKAR3-Ty1) was expressed under the control of a tetracycline repressor in the *Trypanosoma brucei Δpkar1* cell line [7,12]. As expected, expression of LdPKAR3-Ty1 increased in the presence of tetracycline (S5A Fig), with some “leaky” background expression present in the absence of tetracycline. Although the majority of LdPKAR3-Ty1 was found in the DDM-soluble supernatant after detergent fractionation of *T*. *brucei* bloodstream form cells, the protein was also present in the DDM-insoluble pellet (S5B Fig), indicating that the association with the SPMT seen in *L*. *donovani* is preserved upon heterologous expression in *T*. *brucei*. Affinity purification of LdPKAR3-Ty1 from the DDM supernatant using anti-Ty1 antibody coupled to magnetic beads, followed by Western blotting with antibodies against the *T*. *brucei* PKA catalytic subunit, showed that LdPKAR3 formed a stable complex with TbPKAC3 (Fig 4A), but not with TbPKAC1/2 (Fig 4B). Interestingly, up-regulation of LdPKAR3-Ty1 expression was accompanied by increased TbPKAC3 protein levels, possibly due to stabilization when complexed with LdPKAR3 (S5A Fig). Interaction of these subunits was confirmed by co-expression of LdPKAR3 with LdPKAC3 in the *Leishmania tarentolae* LEXSY expression system (Jena Bioscience, Jena) and purification of the heteromeric complex by tandem affinity chromatography using an N-terminal His_6_-tag on LdPKAR3 and a Strep-tag on LdPKAC3 (Fig 4C). As a positive control, we co-purified an LdPKAR1*/*LdPKAC3 complex in a parallel experiment (Fig 4D). We conclude that PKAR3 and PKAC3 form a stable complex similar to the PKAR1/PKAC complexes previously reported in *T*. *brucei* [12], thereby validating PKAR3 as subunit of a PKA holoenzyme complex.

**Fig 5:**
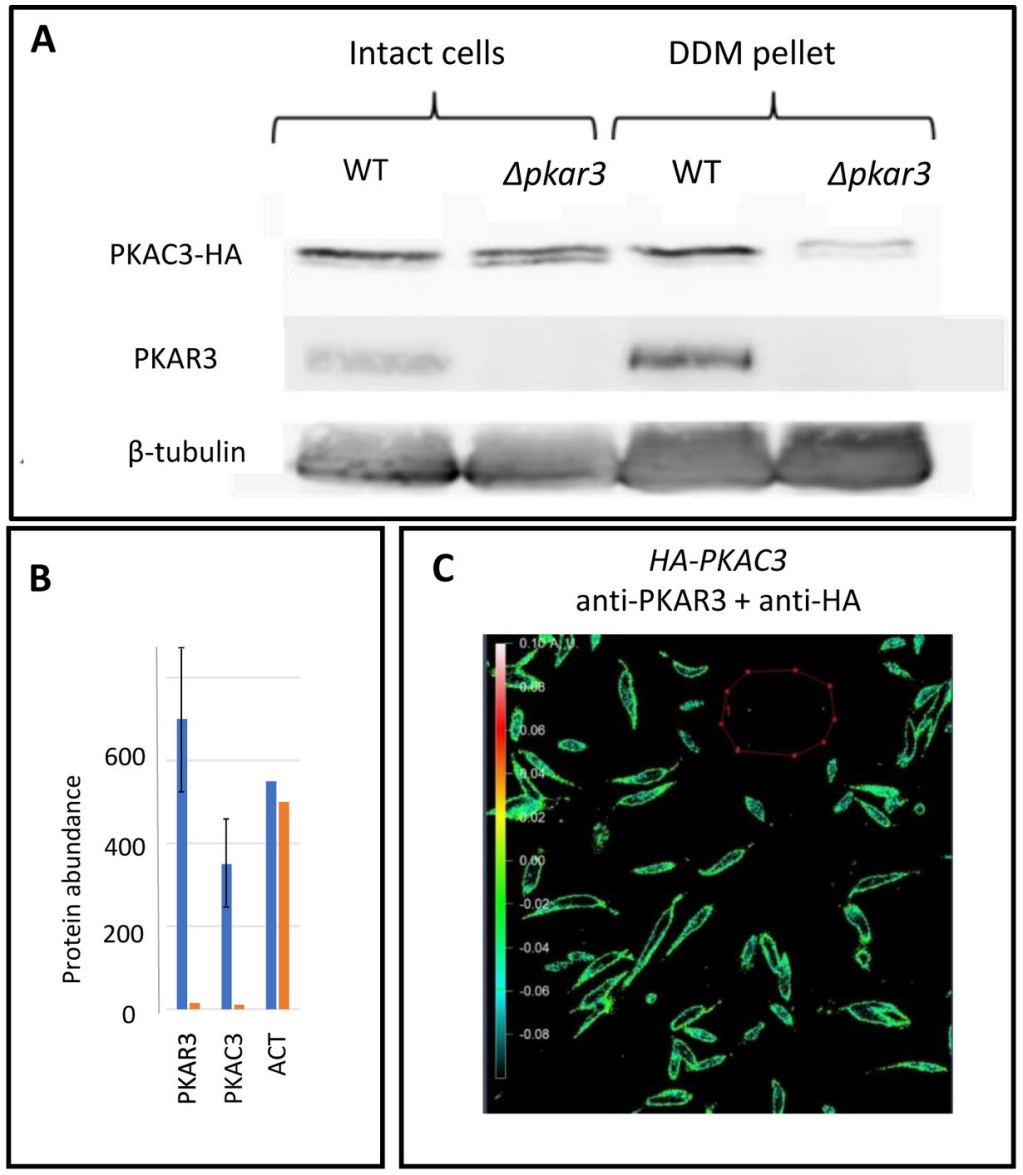
PKAC3 binding to the subpellicular microtubules is mediated by PKAR3. **(A)** Wild type *L*. *donovani* promastigote (WT) and *PKAR3* null mutant (*Δpkar3*) cell lines expressing HA-tagged PKAC3 were subjected to subpellicular microtubule enrichment using 0.5% DDM. Proteins extracted from these were subjected to western blot using antibodies against the HA-tag (upper row), PKAR3 (middle row) and β-tubulin (lower row). The full-length gels are shown in S11 Fig. (**B**) A gel slice containing proteins with a molecular mass range of 35–42 kDa was excised and subjected to mass spectrometry. The mean relative abundance of peptides from PKAC3, PKAR3, and actin (ACT) are shown for experiments using WT (blue bars) and *Δpkar3* (orange bars) cell lines. Error bars show range of n = 2 independent repeats. **(C)** WT cell line expressing HA-tagged PKAC3 was labeled with rabbit anti-PKAR3 and mouse anti-HA, followed by goat anti-rabbit antibodies conjugated to Alexa fluor 568 and goat anti-mouse antibodies conjugated to Alexa fluor 647. The FRET emission intensity was calculated relative to the darkness within the red borders. Emission intensities are color coded (see rainbow ruler on the left) according to values obtained from the Axio Vision program, ranging between -0.01–0.1.

To further assess the interaction between PKAR3 and PKAC3 *in vivo*, HA-tagged PKAC3 was ectopically expressed in *L*. *donovani* WT and *Δpkar3* promastigotes. Western blot analysis of whole cell lysates showed that while PKAC3-HA was expressed at similar levels in both WT and null mutant; it was detected at high levels in the DDM-insoluble fraction from promastigotes of WT, but not *Δpkar3* (Fig 5A). These results were confirmed by proteomic analysis of the DDM-insoluble fraction of WT and *Δpkar3* promastigotes, which showed that the relative abundance of SPMT-associated PKAC3 was ~30-fold higher in WT than *Δpkar3* (Fig 5B). In contrast, actin (which is not associated with PKAR3) showed little difference in relative abundance between samples.

We also employed FRET to confirm the molecular proximity of PKAR3 and PKAC3-HA in *L*. *donovani* promastigotes. As shown in Fig 5C, the high values for corrected acceptor FRET intensity are indicated by yellow to light green pixels at the promastigote cell cortex (-0.02–0 on a scale ranging between -0.08–0.1). The results confirm that PKAR3 and PKAC3 co-localize within the cell cortex in a range compatible with molecular subunit interaction (Figs 3A, 5A and 5B). Promastigotes labeled with antibodies against the donor (PKAR3) or acceptor (PKAC3-HA) alone displayed no background signal (S4C Fig, left and right panels, respectively). Thus, these experiments confirm PKAR3-mediated recruitment of PKAC3 to the SPMT in the parasite cell cortex.

### Structural analysis of the interaction between PKAR3 and PKAC3

**Fig 6:**
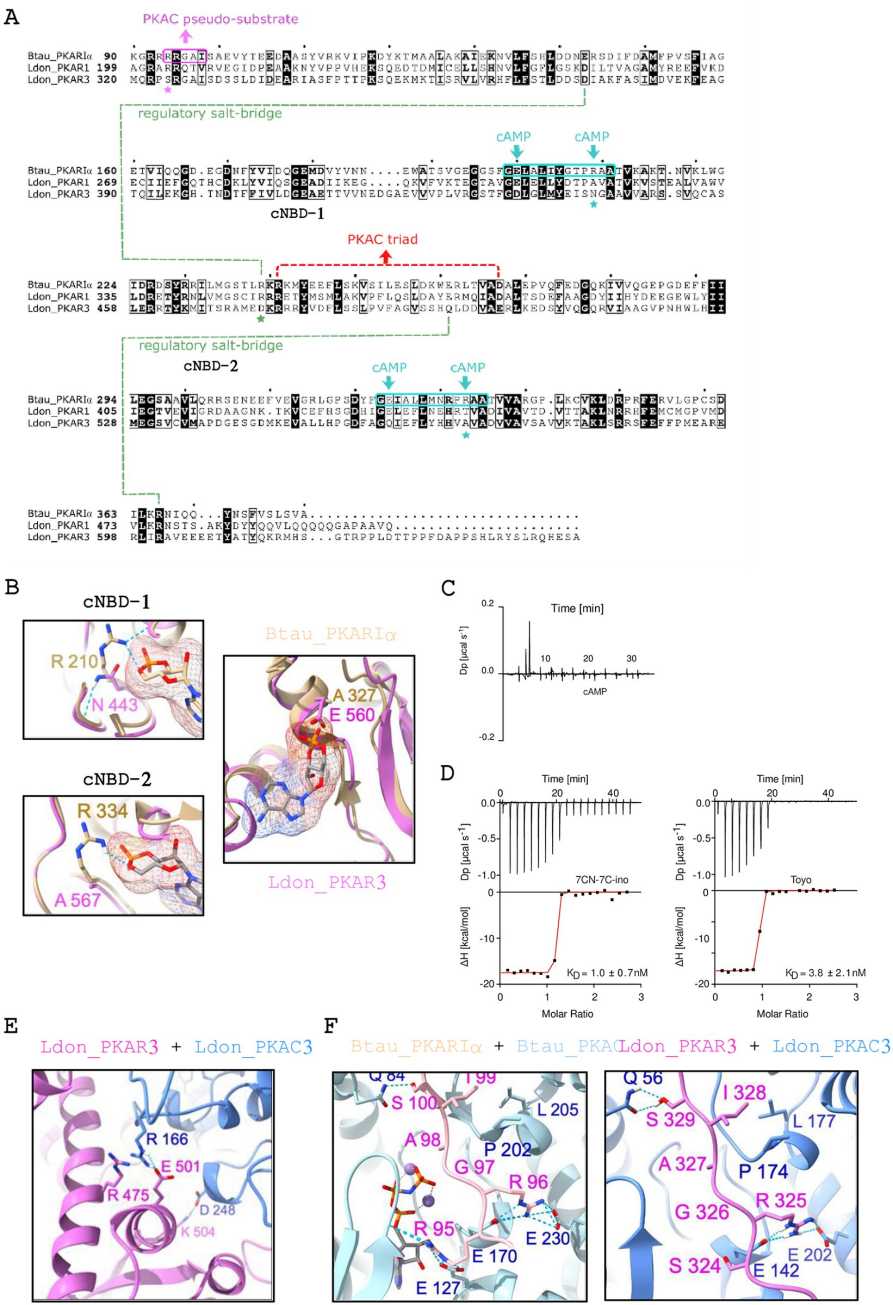
Structural modeling of the PKAR3/C3 complex and ligand binding. (**A**) Protein sequence alignment of *Bos taurus* PKARIα (Btau_PKARIα) with *L*. *donovani* PKAR1 (Ldon_PKAR1) and PKAR3 (Ldon_PKAR3), indicating regions with known structural importance. Amino acids that are conserved in all three sequences are highlighted in black. (**B**) Structural modelling of the two nucleotide binding domains of Ldon_PKAR3 (pink) based on those from Btau_PKARIα (tan), highlighting the most important changes in the conserved amino acid sequence. (**C**) Binding isotherms from isothermal titration calorimetry (ITC) using refolded ligand-free recombinant protein containing the C-terminal portion (residues 321–647) of *L*. *donovani* PKAR3 titrated with cAMP. The graph gives the difference power (DP) between the reference and sample cells upon ligand injection as a function of time. The measurement corresponds to noise, excluding binding of cAMP. The results shown are from a representative example of three independent replicates. (**D**) Similar experiments performed with 7-CN-7-C-inosine (left) or toyocamycin (right). The upper panels show the difference power (DP) between the reference and sample cells after ligand injection as a function of time, while the lower panels show the total heat exchange per mole of injectant (integrated peak areas from upper panel) plotted against the molar ratio of ligand to protein. The K_D_ is the mean of three or more independent replicates, while the graphs are from a single representative replicate. (**E**) Structural model of the complex formed between Ldon_PKAR3 (pink) and Ldon_PKAC3 (blue) showing amino acids predicted to be involved in the salt-bridges and electrostatic interactions downstream of the pseudo-substrate. (**F**) Side chains of critical residues involved in the PKAR-PKAC interaction for experimentally determined Btau_PKARIα/Cα [PDBID:2QCS] (left) and predicted *Ldon_*PKAR3/C3 (right) complexes.

The mammalian PKAR and PKAC subunits have been extensively studied at the atomic level, both alone and in complex [35–37] with an X-ray structure available for *T*. *cruzi* PKAR1 [12], allowing us to perform homology modeling based on these structures. Since the N-terminal sequence of PKAR3 is divergent from human (and other) PKAR subunits and is predicted to be largely low-complexity and disordered (except for the N-terminal 50 amino acids), our models were built using only the C-terminal portion including the inhibitor/pseudo-inhibitor sequence and CNB domains. The conserved residues in the phosphate binding cassettes (PBC) of mammalian CNB domains (R_210_ and R_334_ of PKARIα) are replaced by N_443_ and A_567_ in PKAR3 (Fig 6A and 6B) and E_560_ in the C-terminal binding pocket of PKAR3 likely clashes with the phosphate of cAMP (Fig 6B), as suggested for the *T*. *cruzi* and *T. brucei* PKAR1 crystal structures. We expressed the C-terminal portion of *L*. *donovani* PKAR3 (aa 321–647) in *E*. *coli*, followed by affinity purification, denaturation, and refolding (S6 Fig) to produce ligand-free protein for binding assays by isothermal titration calorimetry (ITC) and to test whether it binds cAMP. These experiments show a complete absence of cAMP binding (Fig 6C), but we found high-affinity binding of the nucleoside analogues 7-cyano-7-deaza-inosine (7-CN-7-C-Ino, also known as Jaspamycin) and Toyocamycin (Toyo), with dissociation constants of 1 nM and 3.8 nM, respectively (Fig 6D); similar to those for the cAMP-independent PKAR1 of *T*. *brucei* [12].

Electrostatic interaction due to salt bridges between conserved amino acids has been shown to be important for interaction between PKAR and PKAC subunits in higher eukaryotes [38]. The salt-bridge triad between R_240_/D_266_ in bovine PKARIα and R_194_ in PKAC that stabilizes this interaction appears to be conserved in *L*. *donovani* PKAR3 (R_475_^/^E_501_) and PKAC3 (R_166_) (Fig 6E). However, non-conservative replacement of the first residue (R_95_ in PKARIα to S_324_ in PKAR3) in the inhibitor/pseudo-inhibitor site is predicted to disrupt interaction with negatively charged and polar residues (T_23_, E_99_, E_142_ and Y_302_) in PKAC3 (Fig 6F). Furthermore, changes from E_143_/R_239_ in PKARIα to D_373_/D_473_ in PKAR3 (Fig 6A) likely prevent formation of a salt bridge critical for cAMP-induced allosteric activation of PKARIα [38]. Thus, the R-C interaction details and ligand-induced conformation changes seem to be significantly different in PKAR3.

### The PKAR3/C3 complex is necessary for maintaining the elongated shape of promastigotes

**Fig 7:**
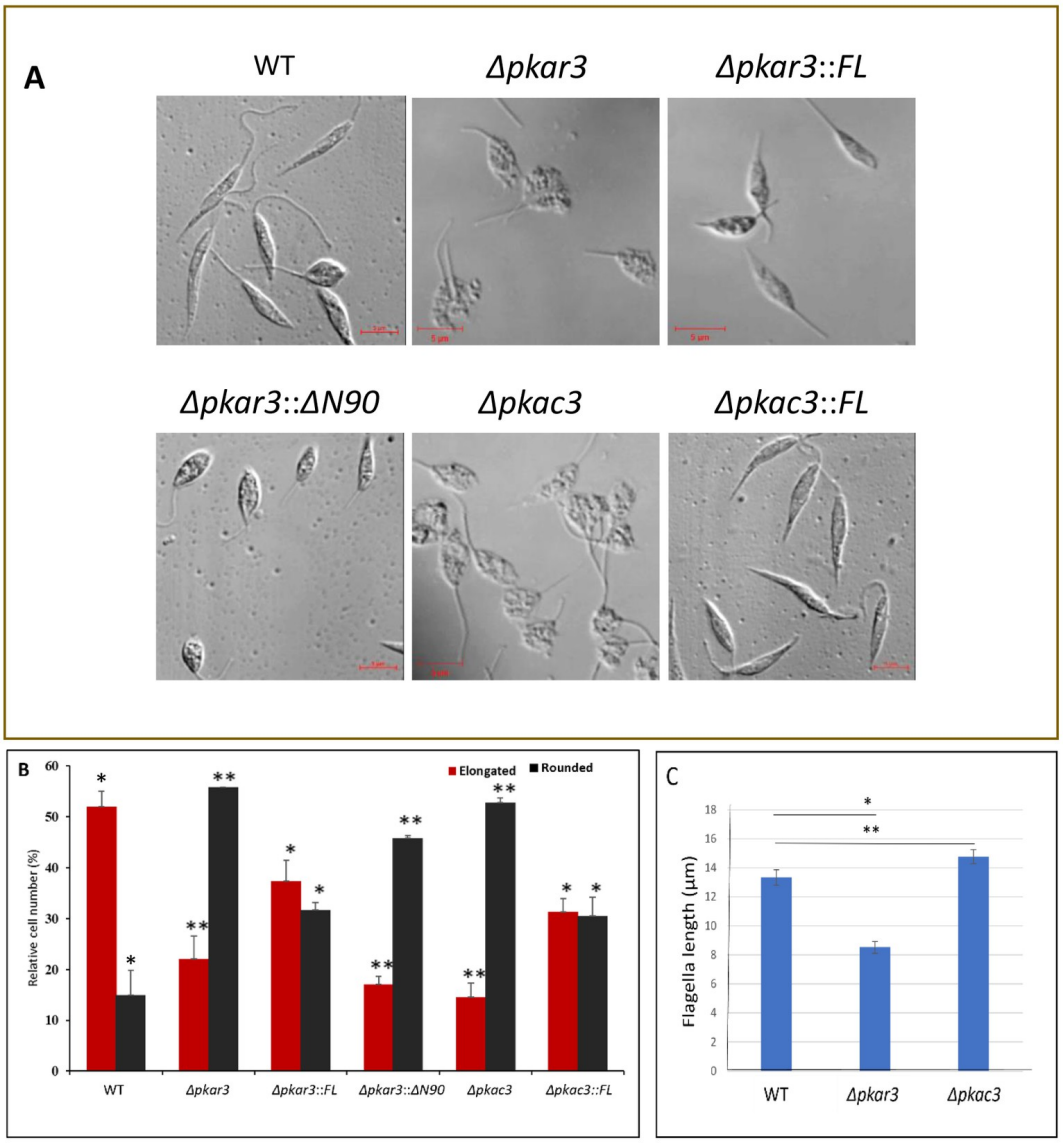
Deletion of *LdPKAR3* or *LdPKAC3* changes elongated *L*. *donovani* promastigote shape to rounded morphology. (**A**) Microscopic DIC pictures of cells from late-log cultures of *L*. *donovani* promastigotes wild type (WT), *PKAR3* null mutant (*Δpkar3)*, *PKAC3* null mutant (*Δpk*ac3), and their add-back derivatives (*Δpkar3*::*FL*, *Δpkar3*::*ΔN90*, and *Δpkac3*::*FL*). **(B**) The percentage of elongated (black bars) or rounded (red bars) cells in the samples above as determined by Image Stream analysis. Ovoid cells (~30% in all cell lines) are not included in the graphs. Asterisks indicate statistically significant differences from WT of p <0.05 (*) or p< 0.01 (**) in three replicate experiments. (**C**) The mean flagellar length of wild type (left bar), *Δpkar3* (middle bar) and *Δpkac3* (right bar), with error bars indicating the range of three independent replicates.

Microscopic examination revealed that a significant number of cells in the *PKAR3* null mutant (*Δpkar3*) population lost the normally elongated shape of wild type (WT) promastigotes and became rounded, although still flagellated (Fig 7A). Ectopic expression of full-length (FL) *PKAR3* largely restored the elongated phenotype, but expression of a truncated (ΔN90) version did not. Similarly, *Δpkac3* promastigotes were more rounded than WT, while ectopic expression of full-length *PKAC3* (partially) restored the elongated shape.

To quantify these changes in shape, we employed Image Streaming flow cytometry [39] to calculate the cell aspect ratio (width/length) of 10,000 cells from each population. Image Stream analysis (S7A Fig) identified three different shape groups in both axenic promastigotes and amastigotes; elongated (aspect ratio of 0.1–0.4), rounded (aspect ratio of 0.6–1), and ovoid (aspect ratio of 0.4–0.6). Elongated cells from WT promastigotes had a mean length of 9.7±0.8 μm with a width of 3.8±0.3 μm, while rounded cells averaged 5.8±1.4 μm long and 4.8±1.1 μm wide (Table 1). All three morphologies are flagellated in promastigotes, with a mean flagellar length of 13.5±1 μm (Table 1), while amastigotes appear aflagellated they retain a short flagellum inside the flagellar pocket. As expected, elongated cells formed the majority (52%) in promastigotes, with only 13% rounded (S7A Fig, upper panels); while axenic amastigotes were largely (78%) rounded, with only 13% elongated (S7A and S7B Fig, lower panels). The relative numbers of ovoid cells (with an aspect ratio between elongated and round) were similar in both promastigotes and amastigotes.

**Table 1:** Image Stream analysis of wild type (WT), *Δpkar3* and *Δpkac3* promastigotes.

	Elongated		Rounded	
	Length	Width	Length	Width
**WT**	9.7±0.8 μm	3.8±0.3 μm	5.8±1.4 μm	4.5±1.1 μm
***Δpkar3***	9.7±1.9 μm	3.7±0.7 μm	5.6±0.1 μm	4.4±0.4 μm
***Δpkac3***	10.9±1.4 μm	3.6±0.5 μm	7.1±0.3 μm	4.2±0.2 μm

The mean cell length and width were determined by Image Stream analysis with >1700 cells counted in each category. The numbers represent the mean value ±SD for three independent samples of each population.

Promastigotes from the *Δpkar3* and *Δpkac3* mutants showed a significantly different distribution of aspect ratio from WT (Fig 7B), with only 22% of *Δpkar3* promastigotes being elongated and the proportion of rounded cells increasing to 56%. Ectopic expression of full-length *PKAR3* in the mutant cell line (*Δpkar3*::*FL)* reduced the fraction of rounded cells to 32%, with the fraction of elongated cells increasing to 37%. In contrast, mutants expressing the *PKAR3*_*ΔN90*_ add-back were similar to the *Δpkar3* parent, with 46% being rounded and only 17% elongated. *PKAC3* null mutants (*Δpkac3*) also showed a substantial increase in the proportion (53%) of rounded cells, with only 14% being elongated. Ectopic expression of full-length *PKAC3* in the *Δpkac3* mutant partially restored the WT phenotype (30% rounded and 31% elongated). Interestingly, Image Stream analysis revealed that while the dimensions of elongated and rounded cells in the *Δpkar3* population were similar to those of WT, those in the *Δpkac3* populations were longer than WT (Table 1). In contrast to fully differentiated amastigotes, rounded promastigotes of *Δpkar3* mutants contain a visible flagellum, although it is considerably shorter than in WT (Fig 7C) with mean flagellar lengths of 8.5±0.8 μm *versus* 13.5±1 μm, respectively. In contrast, the mean flagellar length of *Δpkac3* mutants was slightly longer (15±1 μm) than for WT.

Taken together, the results presented above show that both PKAR3 and PKAC3 are critical for the maintenance of the elongated cell morphology of *Leishmania* promastigotes and that recruitment of PKAC3 to the SPMT by the N-terminal domain of PKAR3 is essential for this process.

## Discussion

**Fig 8:**
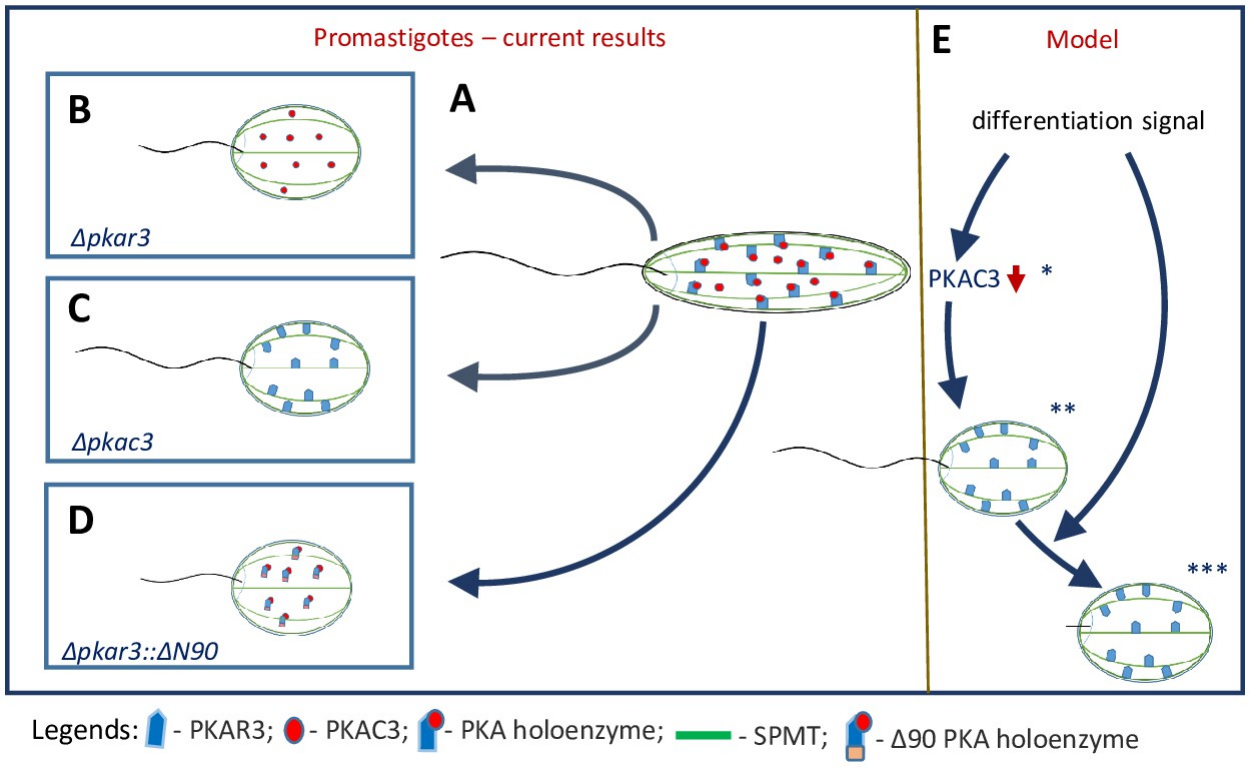
Illustrated summary of conclusions (A-D) and proposed model of differentiation-induced morphogenesis (E). (**A**) PKAR3 recruits PKAC3 to form a holoenzyme at the subpellicular microtubule cortex, thereby maintaining the elongated shape of WT promastigotes. In *Δpkar3* or *Δpkac3* null mutants (**B** and **C**, respectively), the PKAC3 no longer localizes to the SPMT, resulting in rounded promastigotes. Truncation of the putative FH2 domain at the N terminus of PKAR3 (**D**) also releases the PKAR3/C3 complex from the SPMT causing promastigote rounding. (**E**) Model of differentiation induced shape control incorporating published (indicated by asterisks) expression data for PKAC3. PKAC3 is expressed only in promastigotes *[8, 15, 17, 21]; the differentiation signal induces PKAC3 downregulation **[5]. PKA-dependent morphogenesis is only part of the induced differentiation program. Amastigote to elongated promastigote differentiation is reversible ***[7].

In this study, we have functionally characterized a novel regulatory subunit of protein kinase A (PKAR3) that recruits a specific catalytic subunit isoform of PKA (PKAC3) to subpellicular microtubules (SPMT) in the cell cortex of *Leishmania*. This interaction is essential for maintaining the elongated shape of promastigotes, since mutants of *PKAR3* (Δ*pkar3*) become rounded (but remain flagellated), as do those lacking *PKAC3*. Furthermore, truncation of a formin homology (FH2)-like domain at the N-terminal end of PKAR3 resulted in detachment of PKAR3 from the SPMT, again leading to rounded promastigotes. We speculate that PKAC3 phosphorylates one or more proteins in the cell cortex, allowing precise spatiotemporal regulation of microtubule remodeling throughout the parasite’s lifecycle. Fig 8A–8D diagrams the key role of the PKAC3/R3 complex in maintaining promastigote elongation and flagellar length, as deduced from the experiments described above. Interestingly, flagellar shortening occurred in Δ*pkar3* mutants only (Fig 8B and 8D) and thus appears to represent a mechanistically distinct phenotype. A minor fraction of PKAR3 also localizes to the flagellum. where PKAR1 is predominantly located [7, 40]. We speculate that the specific flagellar localization of PKAR3, weakly interacting with the remaining PKAC isoforms, may be responsible for the flagellar length phenotype.

In fission yeast, PKA activity triggered by glucose starvation promotes microtubule destabilization by phosphorylation of the microtubule rescue factor CLASP/Cls/Peg1, although this process apparently does not involve recruitment by PKAR [41]. Microtubule-associated PKA has also been shown to play a critical role in elongation of axons and dendrites by mammalian neurons [42], with PKARIIβ being responsible for the former and PKARIβ for the latter [30]. In this system, microtubule-associated protein 2 (MAP2) binds the D/D domain of the PKARs to link them to the microtubules of neurons [43]. *Leishmania* lacks an obvious orthologue of MAP2, so it appears that the N-terminal FH2-like domain of PKAR3 plays a similar role by recruiting the PKAC3/R3 complex to microtubules, although whether it binds directly or connects *via* an unidentified anchoring protein remains to be seen.

Homologues of PKAC and PKAR are present in most (but not all) eukaryotes [13], with the canonical PKAR containing an N-terminal D/D domain followed by a linker with the inhibitory sequence and two C-terminal cAMP binding domains. The D/D domain typically dimerizes to form a four-helix bundle that serves as a docking site for different A-kinase-anchoring proteins (AKAPs) that allow the PKAR/C complex to be localized to specific sites in the cell. However, PKAR subunits lacking a D/D domain appear several times during eukaryotic evolution [13] and in trypanosomatids PKAR1 contains an RNI-like LRR domain, while PKAR3 contains a long, mostly disordered, N-terminal region. Interestingly, *Paratrypanosoma confusum* (a trypanosomatid parasite isolated from mosquitoes) has an additional paralogue of PKAR (see S1 Table) that is more closely related to the “canonical” PKARs in higher eukaryotes (S1 Fig). All three paralogues (PKAR1, PKAR3 and a “canonical” PKAR) are also found in *Bodo saltans* (a free-living kinetoplastid), suggesting that PKAR1 and PKAR3 arose by gene duplication (and subsequent domain shuffling) early in the evolution of the Kinetoplastidae. Interestingly, PKAR3 has been lost in several trypanosomatid genera; being absent from *Phytomonas* and *Vickermania* (monoxenous insect and/or plant pathogens), as well as four sub-genera (*Duttonella*, *Herpetosoma*, *Nannomonas* and *Trypanozoon*) of *Trypanosoma* (extracellular parasites of mammals). Since many of these species appear to lack amastigotes, this pattern is consistent with the hypothesis that PKAR3 plays an important, but probably not critical, role in regulation of cell morphology during differentiation. However, we cannot preclude the possibility that other trypanosomatids may have developed alternative regulatory mechanisms that do not require PKAR3.

It has been previously shown that sequence changes in the CNBDs of *T*. *brucei* PKAR1 render it refractory to cAMP binding [12, 61]. Here we show that *Leishmania* PKAR3 is equally refractory to cAMP binding and that two high-affinity ligands of PKAR1 (7-CN-7-C-ino and toyocamycin) also bind PKAR3 with affinities in the nanomolar range. However, attempts to dissociate the PKAC3/PKAR3 complex using 7-CN-7-C-ino treatment were unsuccessful, leaving it open whether PKAR3 is a ligand-controlled inhibitor of PKAC3. Indeed, the first loop of the pseudo-inhibitor domain of PKAR3 contains a serine instead of the arginine conserved in other PKARs, suggesting that it cannot dock the PKAC3 active site. Accordingly, PKAC3 may remain catalytically active while complexed with PKAR3, allowing maintenance of the elongated shape of promastigotes. Active PKAC/R complexes have also been shown in higher eukaryotes [30]. It is possible that *Leishmania* (and other Kinetoplastidae) utilize PKAR3 primarily as an anchoring or tethering subunit to localize a specific PKA catalytic subunit close to its target(s) in the cell. In contrast, PKAR1 seems to tether PKAC1/2 to the flagellum in *Leishmania* promastigotes and in *Trypanosoma* [7,18–20], where it responds to nucleoside ligands by subunit dissociation and kinase activation, at least in *T*. *brucei* [12, 61]. The upstream pathway(s) that may signal *via* these nucleoside ligands remain to be identified.

Phosphorylation of the PKAC3/R3 complex may also be involved in regulation of its kinase activity. Indeed, PKAR3 is heavily phosphorylated 2.5 hours after induction of differentiation at multiple sites [8] and PKAC3 is transiently phosphorylated at S_315_ upon induction of differentiation [8, 17]. Alternatively, temporal control of PKA activity may be exerted *via* the steady-state level of PKAC3, with the role of PKAR3 limited to the tethering function, *i*.*e*., the spatial control, as discussed above. This is an attractive speculation, since it has been shown that PKAC3 mRNA (previously named c-lpk2) is reduced by heat (a partial differentiation signal) with very fast kinetics, in part by accelerated decay [15]. In previous studies, we showed that exposure to the full differentiation signal also induces a rapid decrease in PKAC3 mRNA and protein levels [8,17,21,44]. Thus, down-regulation of PKAC3 may be responsible for the rounding observed after exposing promastigotes to the differentiation signal (Fig 8E).

In conclusion, we have shown that *Leishmania* (and likely other trypanosomatid parasites) have re-purposed PKA for a cAMP-independent mechanism of spatiotemporal regulation of microtubule remodeling and of cell shape during parasite development upon host infection.

## Materials and methods

### Phylogenetic analysis of PKA regulatory subunits

The protein sequences of PKARs from selected kinetoplastids (S1 Table) and other eukaryotes (S2 Table) were aligned using the Clustal Omega [45] of Geneious. Phylogenetic trees were constructed using the RAxML module implemented in Rapid Bootstrapping mode with 100 trees using the GAMMA BLOSUM62 substitution matrix [46]. The amino acid sequences of PKAR orthologues were obtained from 19 kinetoplastid genomes representing 17 different genera and two unclassified Trypanosomatidae, as well as eight other eukaryotic species. In several cases, tBlastn searches of unannotated genomes were performed to identify the open reading frame(s) encoding PKAR proteins. Protein domains within each sequence were identified using the InterProScan [47] module of Geneious Prime (https://www.geneious.com) and/or the HHpred website of the MPI Bioinformatics Toolkit [31,32].

### Cell culture

Axenic promastigotes of *L*. *donovani* MHOM/SD/00/1S were grown and maintained as previously described [28,48] in Earle’s-based medium 199 (M199; Biological Industries) supplemented with 10% heat-inactivated Fetal Bovine Serum (GIBCO) and 1% Penicillin-Streptomycin solution (Biological Industries). *Trypanosoma brucei* were cultivated and maintained as described previously [49]. Briefly, bloodstream forms were cultivated at 37°C and 5% CO_2_ in a modified HMI-9 culture medium supplemented with 10% heat-inactivated fetal bovine serum. Cell density was kept below 1×10^6^ cells/ml by regular dilution.

### Detergent enrichment of subpellicular microtubules

Axenic *L*. *donovani* promastigote (20 ml of cells at 1×10^7^ cells/ml) were washed twice with ice-cold phosphate buffered saline (PBS). The final pellet was suspended to 200 μl PBS containing protease inhibitors mix (Roche cOmplete protease inhibitor) and 0.5% n-dodecyl β-D-maltoside (DDM) in Eppendorf tubes. After a short vortex, the tubes were placed on ice for 15 min, and subsequently centrifuged. The pellet was washed twice with 1 ml of ice-cold PBS containing the protease inhibitors. For immunofluorescence analysis, the pellet was suspended in 100 μl PBS. For western blot analysis, the pellet was suspended in 100 μl Laemmli buffer. *T*. *brucei* subpellicular microtubules were enriched employing the same protocol used for *Leishmania*.

### Generation of a polyclonal anti-PKAR3 antibody

Recombinant PKAR3 was purified from transgenic *Escherichia coli* ectopically expressing PKAR3 as we have done previously [24]. Briefly, the full-length CDS of R3 (encoded by *LinJ*.*34*.*2680*) was polymerase chain reaction (PCR) amplified from genomic DNA of *L*. *infantum* strain JPCM5 using primers (S3 Table) designed to introduce an N-terminal His_6_ tag followed by a TEV protease cleavage site and cloned *via Bam*HI and *Not*I restriction sites into pETDuet-1 (Novagen, Merck Millipore). The protein was expressed in *E*. *coli* Rosetta and purified using Ni-NTA columns followed by TEV protease cleavage. Recombinant R3 was injected to two rabbits with 5 mg/ml protein each. Rabbits were boosted four times with 5 mg/ml of the recombinant PKAR3. Injections and bleedings were carried out as a contracted service (Sigma-Aldrich).

### Western blotting

Western blot analysis was done as described previously [48], using a 1:1,000 dilution of polyclonal rabbit anti-PKAR3, anti-TbPKAR1 [7], anti-TbPKAC1/C2 [12] and anti-TbSAXO [25]. Rabbit antibodies against TbPKAC3 were used at 1:250 dilution [12]. For western blot detection of proteins recombinantly expressed in *L*. *tarentolae*, mouse anti-His (Bio-Rad) and mouse anti-Strep (Qiagen) were used. The secondary antibodies IRDye680LT goat anti-rabbit (1:50,000; LICOR) and IRDye800CW goat anti-mouse (1:10,000; LICOR) were used for detection with the Odyssey CLx imaging system (LICOR). Rabbit antiserum against *L*. *donovani* HSP83 [28] or β-tubulin (Cell Signalling) were used as protein loading markers.

### Gene knockouts

The *PKAR3 and PKAC3* genes were deleted from the 1S-2D clone of *L*. *donovani* using homologous recombination as described previously by Inbar et al. [34]. PCR analyses of genomic DNA were used to confirm the absence of the targeted genes (S2B Fig). Polyclonal antibodies raised in rabbits against recombinant PKAR3 reacted with a protein of the correct molecular mass (72 kDa) in western blots of wild type cells, but not *Δpkar3* mutants (S2A Fig). The protein was present (at slightly elevated levels) in add-back mutants (*Δpkar3*::FL) ectopically expressing (in an episome) full-length PKAR3. The *PKAC3* gene was deleted using the same strategy as above. PCR analyses of *L*. *donovani* genomic DNA extracted from single colonies confirmed that both copies of the gene are missing from *Δpkac3*, while the antibiotic resistance genes that were inserted to replace *PKAC3* are in place (S8 Fig).

### Immunofluorescence

Indirect immunofluorescence analyses were carried out following the methods described by Inbar et al. [34]. Briefly, late log phase promastigotes were washed twice in PBS and then fixed in 1% formaldehyde/PBS on a slide for 10 min before permeabilization by exposure to 0.2% TritonX-100/PBS (PBST) for 10 min. Cells were incubated with blocking solution [5% (v/v) non-fat dried skimmed milk powder/PBST] for 30 min at room temperature, incubated with rabbit anti-PKAR3 (this study), rabbit anti-promastigote membrane proteins [28] or mice anti-HA tag antibodies (1:500 dilution) for one hour. Subsequently, slides were washed three times with PBS-Tween and incubated with the fluorescent secondary antibody in darkness for 30 min. Slides were washed three times with PBS-Tween and a drop of DAPI Fluoromount G (Southern Biotech) was added. Slides were covered with slips, sealed, and then kept in darkness. Subsequently, they were examined using a Zeiss LSM 700 inverted confocal laser scanning microscope. Image processing was done using Zen lite software, Zeiss.

### Fluorescence Resonance Energy Transfer (FRET)

Late log phase *L*. *donovani* promastigotes ectopically expressing PKAR3 and PKAC3-HA were fixed in PBS containing 4% paraformaldehyde and then settled on slides. PKAR3 was labelled using polyclonal anti-PKAR3 antibody (1:500), followed by detection with goat anti-rabbit Alexa 568 (1:500). PKAC3 was labelled with anti-HA antibody (1:1000, BioLegend) and secondary goat anti-mouse Alexa 647 (1:500). Confocal microscopy was obtained using a Zeiss LSM 700 Inverted confocal laser scanning microscope. An excitation wavelength of 578 nm and an emission wavelength of 640 nm and below were used for Alexa568, whereas an excitation wavelength of 651 nm and an emission wavelength of 640 nm and above were used for Alexa647. FRET was assessed with AxioVision FRET software, using the mathematical approach—Youvan’s technique. Youvan’s technique (Fc) is the basis of all correction measurement techniques (out of 4 techniques) in AxioVision FRET. It refers to corrected FRET values when three FRET filter sets are used. The measured values are corrected for the background (bg) and for the crosstalk from the donor (don) and the acceptor (acc). Calculation of the intensity values for FRET follows the formula: Fc = (fretgv—bg)—cfdon (dongv—bg)—cfacc (accgv—bg), where gv is the fluorescence value in every pixel and cf is a constant, which is calculated for each of the samples with only one secondary antibody. Subsequently, the number of interacting molecules in the FRET images were counted using the microscopic analysis software, IMARIS version 9.3.

### Image Stream flow cytometry

The Image streamer was calibrated by feeding it with late log phase axenic promastigotes of wild type and mutants. Parasites were washed once using ice cold phosphate buffered saline (PBS). Subsequently, cells were fixed by suspending them in PBS containing 4% paraformaldehyde. An aliquot of these cell suspension was injected into the Image Stream Mark II. Each cell was subjected to a snapshot in bright field. The data was then analysed using IDEAS software, version 6.10, with compensation according to the software standards [50–52]. Cells were gated for single cells in focus (S6 Fig). Further, cells were classified based on shape (elongated, ovoid, rounded) by considering aspect ratio and symmetry features. The aspect ratio assesses the morphology of cells by calculating width/length. Symmetry 2 measures the tendency of the object to have single axis of elongation.

### Flagella length measurements

Late log phase *L*. *donovani* promastigotes of wild type, Δ*pkaR3* and Δ*pkaC3* were aliquoted and then fixed as done for the preparation for image streaming analyses. DIC images of each culture were used to measure the flagella length of 200 cells using the imageJ software [53].

### Inducible overexpression of PKAR3 in *Trypanosoma brucei*

The *PKAR3* CDS was PCR amplified from genomic DNA of *L*. *donovani* promastigotes using primers R3_HindIII_fw and R3_lr_Ty1_BamHI_rev (S3 Table) to introduce a C-terminal Ty1 tag. The PCR product was cloned into the *Hin*dIII and *Bam*HI sites of pHD615 [54], which was then linearized with *Not*I for transfection of a previously described homozygous *T*. *brucei PKAR1* deletion mutant [7] that additionally expresses a double tetracycline repressor (pHD1313; [55]. Transfected cells were selected with 2 μg/ml blasticidin (pHD615] and 2.5 μg/ml phleomycin (pHD1313). PKAR3 expression was induced with 1 μg/ml tetracycline.

### Immunoprecipitation in *T*. *brucei*

Immunoprecipitation of PKAR3-Ty1 was carried out by binding anti-Ty1 [56] to magnetic protein A beads (Dynabeads, Invitrogen) followed by a 2-hour incubation with 1×10^8^ trypanosomes lysed in lysis buffer (10 mM Tris/Cl pH 7.5; 150 mM NaCl; 0.5 mM EDTA; 0.5% NP-40; Roche cOmplete protease inhibitor) for 30 min at 4°C. Beads were washed 4x with lysis buffer and proteins were eluted by incubation with 50 μl 2× Laemmli sample buffer for 5 min at 95°C.

### Tandem affinity purification from *Leishmania tarentolae*

PKAR1 and PKAR3 were N-terminally tagged with a hexa-histidine peptide by PCR amplification from *L*. *donovani* genomic DNA. The PCR products were inserted in the pLEXSY_I-ble3 vector (Jena Bioscience). PKAC3 was N-terminally strep-tagged and inserted into pLEXSY_I-neo3. The vectors were then linearized with *Swa*I and stably co-transfected for holoenzyme expression (PKAR1 and PKAC3 or PKAR3 and PKAC3) into the LEXSY T7-TR cell line (Jena Bioscience). Protein co-expression, parasite cultivation and protein purification were performed as described in [12] with a few modifications. Briefly, co-expression was induced with 10 μg/ml tetracycline for 48 hours. The cells were harvested by centrifugation (2000×g for 5 min), washed with PBS, and lysed in His binding buffer (50 mM NaH_2_PO_4_ pH 8, 300 mM NaCl, 10 mM imidazole, 1% Triton-X, protease inhibitor cocktail). The soluble fraction was loaded onto a gravity flow Ni-NTA column (Thermo Fisher Scientific). The column was washed twice with His wash buffer (50 mM NaH_2_PO_4_ pH 8, 300 mM NaCl, 20 mM imidazole) prior to elution of the kinase complex with His elution buffer (50 mM NaH_2_PO_4_ pH 8, 300 mM NaCl, 250 mM imidazole). The Ni-NTA eluate was loaded on to a gravity flow Strep-Tactin column (IBA), which was then washed with Strep wash buffer (50 mM NaH_2_PO_4_ pH 8, 300 mM NaCl). Lastly, the kinase complexes were eluted with Strep elution buffer (50 mM NaH_2_PO_4_ pH 8, 150 mM NaCl, 50 mM biotin). The integrity of the co-expressed holoenzyme complexes was analysed by western blot.

### Pulldown of subpellicular microtubules-bound proteins (proteomics)

These experiments aimed to determine the levels of PKAC3 binding to the subpellicular microtubules of *L*. *donovani* wild type (WT) and *Δpkar3*. Microtubules of WT and *Δpkar3* promastigotes were DDM-enriched as described above. Subsequently, proteins associated with this fraction were separated on 9% SDS-PAGE and a slice containing proteins at the molecular mass range of 35–42 kDa was excised and subsequently subjected to mass spectrometry. The proteins in the gel were reduced with 3 mM DTT (60°C for 30 min), modified with 10 mM iodoacetamide in 100 mM ammonium bicarbonate (in the dark, room temperature for 30 min) and digested in 10% acetonitrile and 10 mM ammonium bicarbonate with modified trypsin (Promega) at a 1:10 enzyme-to-substrate ratio, overnight at 37°C. The resulting peptides were desalted using C18 tips (Homemade stage tips), dried and resuspended in 0.1% formic acid. The peptides were resolved by reverse-phase chromatography on 0.075 X 180-mm fused silica capillaries (J&W) packed with Reprosil reversed phase material (Dr Maisch GmbH, Germany). The peptides were eluted with linear 60 minutes gradient of 5 to 28%, 15 minutes gradient of 28 to 95% and 15 minutes at 95% acetonitrile with 0.1% formic acid in water at flow rates of 0.15 μl/min. Mass spectrometry was performed by Q Exactive plus mass spectrometer (Thermo) in a positive mode using repetitively full MS scan followed by collision-induced dissociation (HCD) of the 10 most dominant ions selected from the first MS scan. The mass spectrometry data was analyzed using Proteome Discoverer 1.4 software with Sequest (Thermo) algorithm against *L*. *donovani* and *L*. *infantum* proteomes from TriTryp database with mass tolerance of 20 ppm for the precursor masses and 0.05 Da for the fragment ions (TriTrypDB). Oxidation on Met, and Phosphorylation on Ser, Thr, Tyr were accepted as variable modifications and Carbamidomethyl on Cys was accepted as static modification. Minimal peptide length was set to six amino acids and a maximum of two miscleavages was allowed. Peptide- and protein-level false discovery rates (FDRs) were filtered to 1% using the target-decoy strategy. Semi-quantitation was done by calculating the peak area of each peptide based on its extracted ion currents (XICs) and the area of the protein that is the average of the three most intense peptides from each protein. The mass spectrometry proteomics data have been deposited to the ProteomeXchange Consortium via the PRIDE [55] partner repository with the dataset identifier: PXD025222.

### Molecular modelling

PKAR3 homology models were built from each of the PDB templates 2QCS, 5JR7 and 4MX3 with Chimera Modeller [57, 58]. Multiple sequence alignments were produced with Clustal Omega [45]. The PKAR3/C3 complex was predicted by AlphaFold2 using MMseqs2 on ColabFold v1.5.2, with pdb70 templates, alphafold2_multimer_v3 weights for complex predictions using 20 recycles and tolerance set to 0.5, followed by AMBER relaxation [59]. Structure images were produced with UCSF Chimera [60].

### Ligand binding studies by isothermal titration calorimetry (ITC)

Protein purification was performed as described in [12] with the following modification: N-terminally truncated LdPKAR3 (aa 321–647) was cloned into pETM11_Sumo3 (NEB) with an N-terminal Sumo3-tag and expressed in *E*. *coli* Rosetta (DE3). Purification of R3(321–647) by Ni-NTA affinity chromatography was followed by SenP2 protease-mediated cleavage of the N-terminal Sumo3-tag during dialysis of the protein in 50 mM HEPES pH 7.5 and 50 mM NaCl. The cleaved protein was either stored at -80°C or directly denatured and separated from prebound ligands as described [12]. For ligand binding studies by ITC the protein was further dialyzed against 50 mM Tris-Cl pH 8.5, 9.6mM NaCl, 0.4 mM KCl, 2 mM MgCl2, 2 mM CaCl2, 0.5 M Arginine, 0.75 M Guanidine-HCl, 0.4 M Sucrose, 10 mM DTT overnight at 4°C and subsequently purified by gel filtration chromatography on a Superdex Increase 10/300 GL column (GE healthcare). Refolded and purified proteins were diluted to 10–20 μM in ITC buffer (50 mM HEPES pH 7.5 50 mM NaCl and 1% DMSO). 100–200 μM 7-cyano-7-deaza-inosine (7-CN-7C-ino) or Toyocamycin (Toyo) was diluted in the same buffer. ITC measurements were carried out on a MicroCal PEAQ-ITC (Malvern instrument). 2–4 μl of ligand were injected in a series of 13–19 injections into the protein sample at 298K. The Differential Power (DP) between the reference and sample cell was maintained at 8–10 μcal s–^1^ in all experiments. Data analysis was performed with the MicroCal PEAQ-ITC software applying a model with one binding site.

## Supporting information

S1 Fig Phylogenetic analysis of Kinetoplastae PKARs. Fifty-one PKAR proteins from 19 kinetoplastid genomes representing 17 different genera and two unclassified Trypanosomatidae, as well as eight other eukaryotes, were aligned using Clustal Omega and an unrooted phylogenetic tree constructed by RAxML bootstrapping. Proteins containing D/D domains are indicated in red (canonical) or orange (non-canonical), while those containing N-terminal LRRs are shown in green. The blue box indicates the PKAR orthologues from *Bodo saltans* and *Paratrypanosoma confusum* clustering with the “canonical” PKARs from other eukaryotes.
(TIF)

S2 Fig Deletion of *PKAR3* from the genome of *L*. *donovani* promastigotes. (**A**) Proteins extracted from *L*. *donovani* WT (left lane), *Δpkar3* (middle lane) and *Δpkar3* ectopically expressing the full-length LdPKAR3 (*Δpkar3*::FL; addback; right lane) were subjected to western blot using the antibodies raised against LdPKAR3. β-tubulin was used as a loading control. (**B**) PCR on genomic DNA extracted from promastigotes of WT (FL) and homozygous *Δpkar3*. *PKAR3* ORF (upper panel) is seen only on DNA extracted from WT cells, while the mutant contains both resistance marker genes, hygromycin (Hyg) and neomycin (Neo).
(TIF)

S3 Fig HHpred analysis of *Leishmania* PKAR3. The N-terminal 100 amino acids of *L*. *donovani* PKAR3 were submitted to the HHpred server, with the best matches in the Protein Data Bank (PDB) shown in the upper panel. A description of the top 12 hits is shown in the lower panel. Proteins containing formin homology (FH2) domains are indicated by red arrows.
(TIF)

S4 Fig FRET analysis between PKAR3 and β-tubulin and PKAR3 and PKAC3 –control experiments. **(A)** *PKAR3* null mutant (*Δpkar3*) labeled with the same antibodies as in panel (A) of Fig 3. FRET is shown in the left panel and immunofluorescence in the right panel. (**B)** WT promastigotes labeled with rabbit antibodies against the AAP24 transporter and mouse antibodies against β-tubulin, followed by secondary antibody detection with goat anti-rabbit antibodies conjugated to Alexa Fluor 568 and donkey anti-mouse antibodies conjugated to Alexa Fluor 647. FRET emission is shown in the left panel and immunofluorescence in the right panel. (**C**) The two panels show controls for the FRET between PKAR3 and PKAC3 as in Fig 5C using antibody against PKAR3 (donor) or HA-tagged PKAC3 (acceptor) alone, followed by the two secondary antibodies together as in the left panel. Significant FRET emission indicates interaction between the two labeled proteins.
(TIF)

S5 Fig Controls for LdPKA subunit interaction. LdPKAR3 with a C-terminal Ty1-tag was inducibly (+/- Tet) expressed in *T*. *brucei* MiTat 1.2 Δ*pkar1* null cells (Δ*tbpkar1* LdPKAR3-Ty1) (2). (**A**) Western blot with anti-PFR-A/C as loading control; LdPKAR3-Ty1 and TbPKAC3 are detected at the expected molecular mass using anti-LdPKAR3 and anti-TbPKAC3 antibodies, respectively. (**B**) DDM extraction with whole cell lysate (WCL), soluble fractions 1 and 2 (sol) and detergent-resistant pellet (P). Part of LdPKAR3-Ty1 and TbPKAC3 remain in the pellet fraction. The microtubule-associated protein TbSAXO (1) serves as marker for the detergent resistant fraction.
(TIF)

S6 Fig Purification of LdPKAR3 recombinantly expressed in *E*. *coli*. Representative size exclusion chromatogram of refolded LdPKAR3(321–647) used for ITC. The collected fraction is marked with a line above the UV-peak. Purity and expected molecular mass are confirmed by SDS-PAGE (inset).
(TIF)

S7 Fig Image streamer calibration with cell shape of axenic *L*. *donovani* promastigotes and amastigotes. **A.** An Amnis Image StreamX Mk II image streamer was fed with either late-log phase axenic promastigotes (upper panel) or amastigotes (lower panel). Snapshot of 10,000 cells per each group was taken and used to calculate the width/length aspect ratio. Cells were classified based on their aspect ratio range as elongated (E, 0.1–0.4) or rounded (R, 0.6–1.0). **B.** Representative snapshots of elongated and rounded flagellated promastigotes (upper panel) and elongated and rounded aflagellated amastigogtes. The %age of each population is indicated.
(TIF)

S8 Fig Deletion of *LdPKAC3* from the *L*. *donovani* genome. PCR on extracted genomic DNA from (**A**) wild type or (**B**) *Δpkac3* cells. The *PKAC3* ORF is detected only in wild type cells, while in *Δpkac3* cells both resistance marker cassettes, hygromycin (Hyg) and neomycin (Neo) are amplified.
(TIF)

S9 Fig Full length of western blot gels in Fig 1C. Experimental details are in the Legend to Fig 1C. Antibodies used; anti PKAR3, β tubulin and HSP83.
(TIF)

S10 Fig Full length of western blot gels in Fig 2. (**A**) the full-length gels of Fig 2A, (**B**) the full-length gels of Fig 2C. Experimental details are in the legends to Fig 2
(TIF)

S11 Fig Full length of western blot gels in Fig 5. Experimental details are in the legends to Fig 5. The full-length gels of Fig 5A.
(TIF)

S1 Table A list of protein kinase A regulatory (PKAR) subunits in the *Kinetoplastidae*. The list of PKAR genes of Kinetoplastidae in this table is from TriTrypDB website (TriTrypDB). GenBank accession numbers refer to the nucleotide sequence containing an open reading frame with a Blast hit to PKAR1 and/or PKAR3.
(DOCX)

S2 Table Protein kinase A regulatory (PKAR) subunits in selected other eukaryotes. The list of PKAR genes is from Gene Databank.
(DOCX)

S3 Table Primers used in this stud. The list of primers and their sequence is provided.
(DOCX)
