# Supplementary figures and images for: A divergent protein kinase A regulatory subunit essential for morphogenesis of the human pathogen Leishmania
Source: PLoS Pathog. 2024 Mar 29;20(3):e1012073. doi: 10.1371/journal.ppat.1012073 (PMC11006142; doi:10.1371/journal.ppat.1012073)

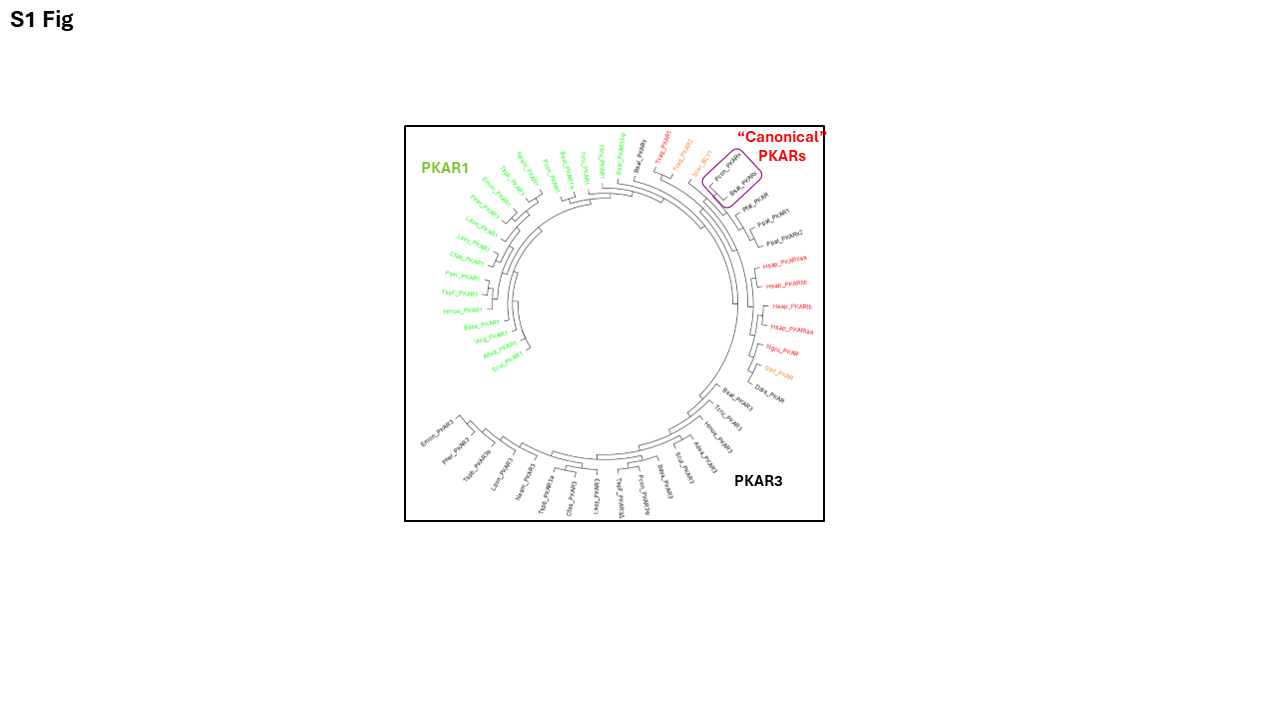

Supplement: S1 Fig — Fifty-one PKAR proteins from 19 kinetoplastid genomes representing 17 different genera and two unclassified Trypanosomatidae, as well as eight other eukaryotes, were aligned using Clustal Omega and an unrooted phylogenetic tree constructed by RAxML bootstrapping. Proteins containing D/D domains are indicated in red (canonical) or orange (non-canonical), while those containing N-terminal LRRs are shown in green. The blue box indicates the PKAR orthologues from Bodo saltans and Paratrypanosoma confusum clustering with the “canonical” PKARs from other eukaryotes. (TIF) [file ppat.1012073.s001.tif]

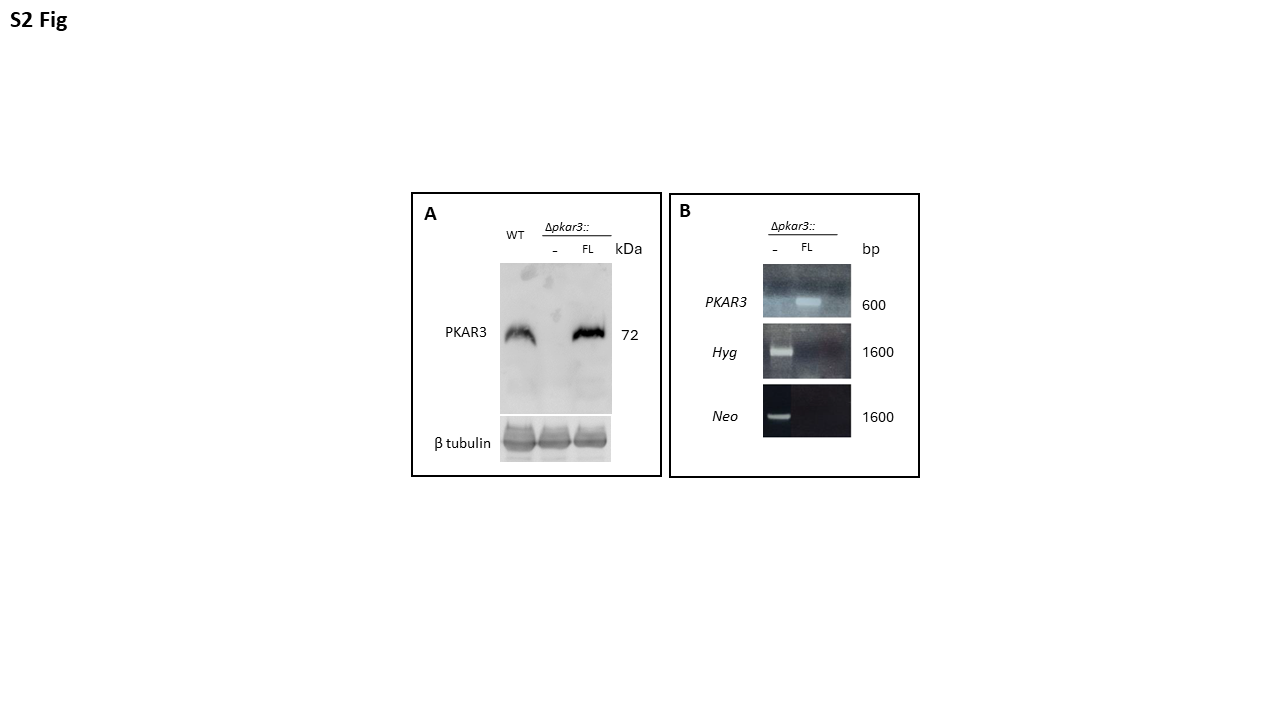

Supplement: S2 Fig — (A) Proteins extracted from L. donovani WT (left lane), Δpkar3 (middle lane) and Δpkar3 ectopically expressing the full-length LdPKAR3 (Δpkar3::FL; addback; right lane) were subjected to western blot using the antibodies raised against LdPKAR3. β-tubulin was used as a loading control. (B) PCR on genomic DNA extracted from promastigotes of WT (FL) and homozygous Δpkar3. PKAR3 ORF (upper panel) is seen only on DNA extracted from WT cells, while the mutant contains both resistance marker genes, hygromycin (Hyg) and neomycin (Neo). (TIF) [file ppat.1012073.s002.tif]

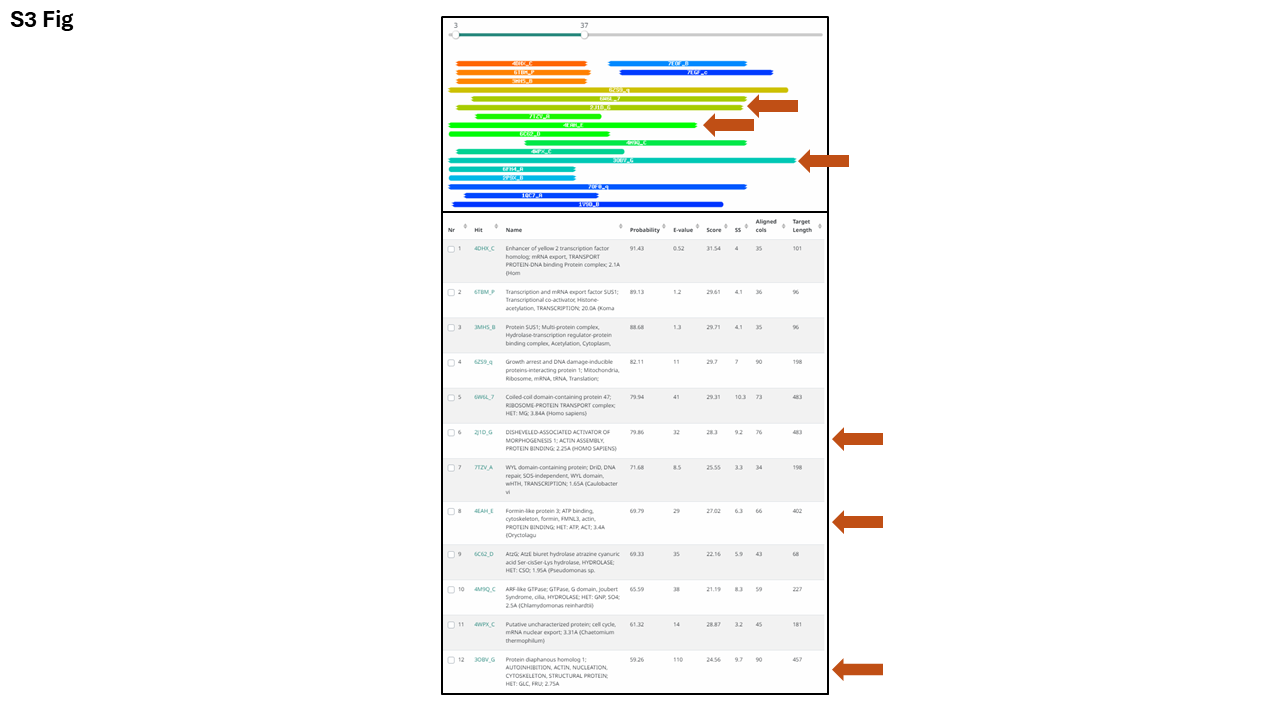

Supplement: S3 Fig — The N-terminal 100 amino acids of L. donovani PKAR3 were submitted to the HHpred server, with the best matches in the Protein Data Bank (PDB) shown in the upper panel. A description of the top 12 hits is shown in the lower panel. Proteins containing formin homology (FH2) domains are indicated by red arrows. (TIF) [file ppat.1012073.s003.tif]

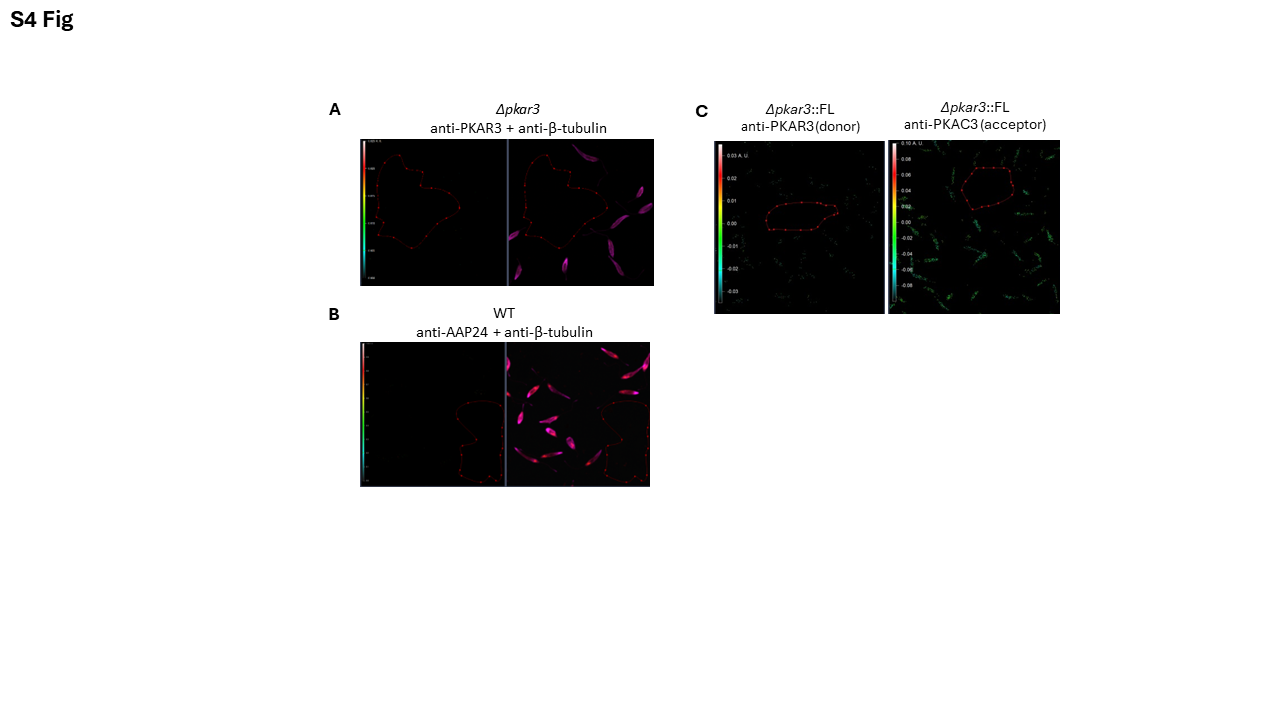

Supplement: S4 Fig — (A) PKAR3 null mutant (Δpkar3) labeled with the same antibodies as in panel (A) of Fig 3. FRET is shown in the left panel and immunofluorescence in the right panel. (B) WT promastigotes labeled with rabbit antibodies against the AAP24 transporter and mouse antibodies against β-tubulin, followed by secondary antibody detection with goat anti-rabbit antibodies conjugated to Alexa Fluor 568 and donkey anti-mouse antibodies conjugated to Alexa Fluor 647. FRET emission is shown in the left panel and immunofluorescence in the right panel. (C) The two panels show controls for the FRET between PKAR3 and PKAC3 as in Fig 5C using antibody against PKAR3 (donor) or HA-tagged PKAC3 (acceptor) alone, followed by the two secondary antibodies together as in the left panel. Significant FRET emission indicates interaction between the two labeled proteins. (TIF) [file ppat.1012073.s004.tif]

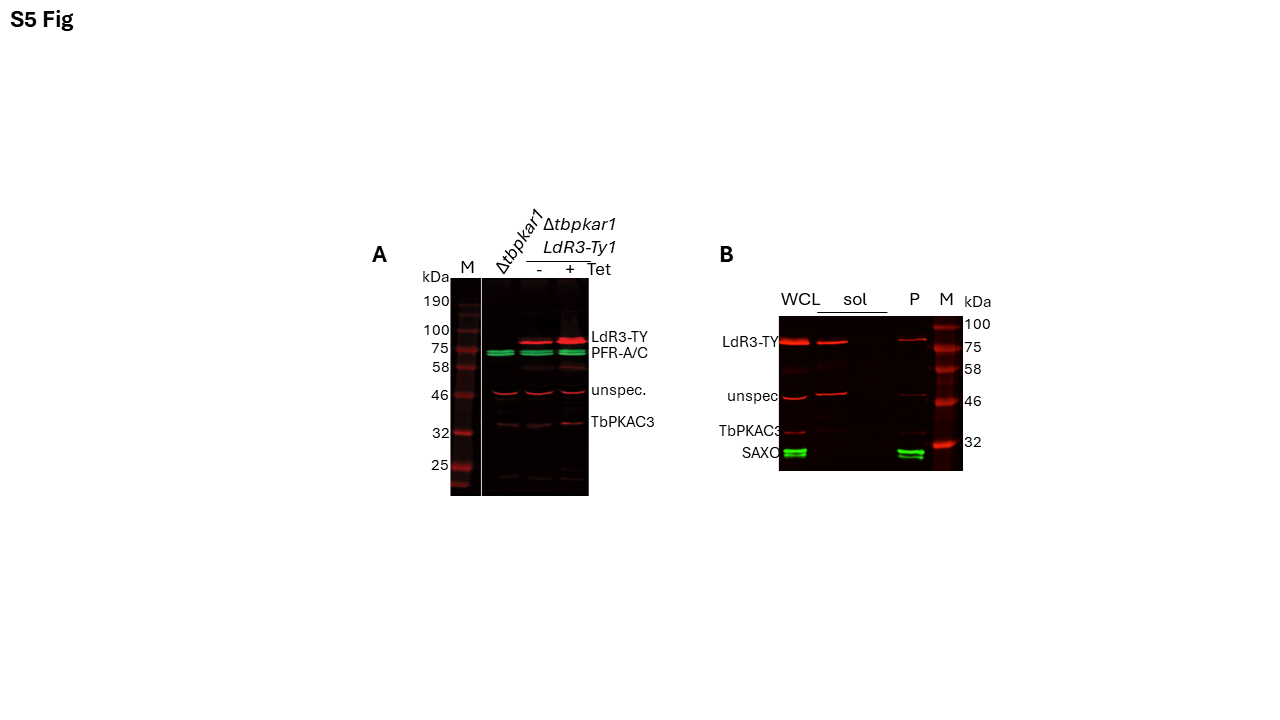

Supplement: S5 Fig — LdPKAR3 with a C-terminal Ty1-tag was inducibly (+/- Tet) expressed in T. brucei MiTat 1.2 Δpkar1 null cells (Δtbpkar1 LdPKAR3-Ty1) (2). (A) Western blot with anti-PFR-A/C as loading control; LdPKAR3-Ty1 and TbPKAC3 are detected at the expected molecular mass using anti-LdPKAR3 and anti-TbPKAC3 antibodies, respectively. (B) DDM extraction with whole cell lysate (WCL), soluble fractions 1 and 2 (sol) and detergent-resistant pellet (P). Part of LdPKAR3-Ty1 and TbPKAC3 remain in the pellet fraction. The microtubule-associated protein TbSAXO (1) serves as marker for the detergent resistant fraction. (TIF) [file ppat.1012073.s005.tif]

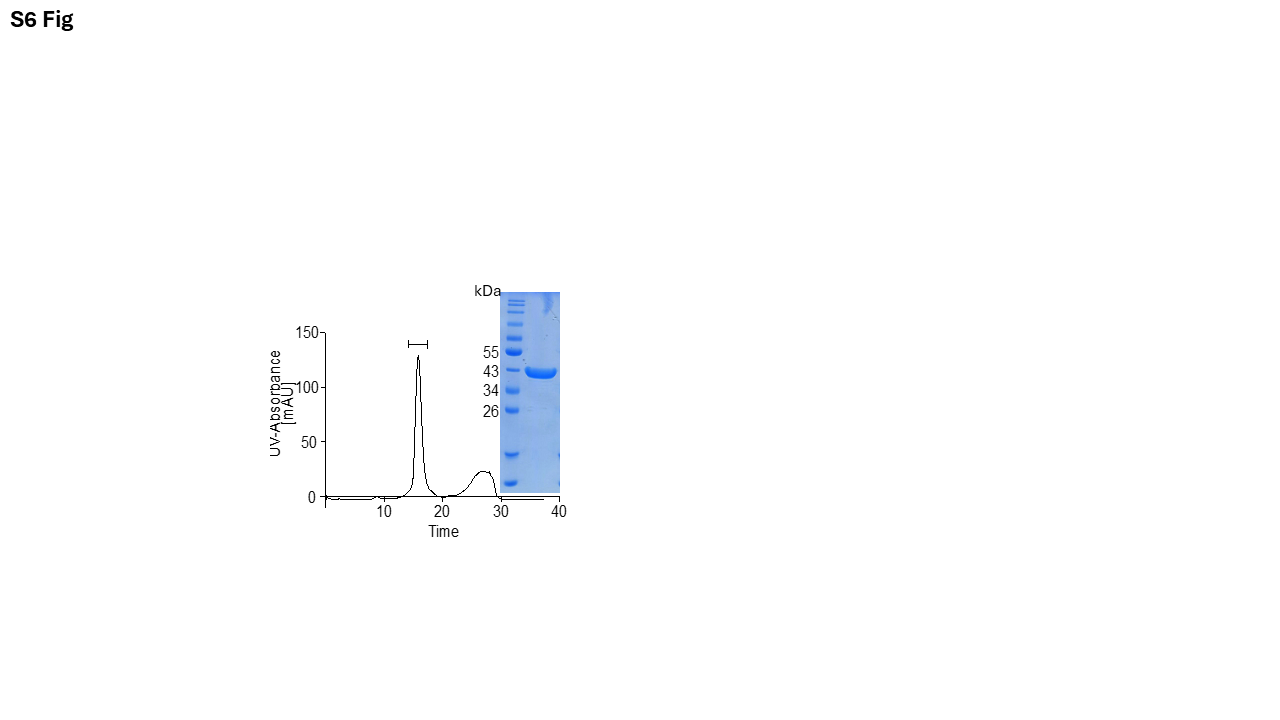

Supplement: S6 Fig — Representative size exclusion chromatogram of refolded LdPKAR3(321–647) used for ITC. The collected fraction is marked with a line above the UV-peak. Purity and expected molecular mass are confirmed by SDS-PAGE (inset). (TIF) [file ppat.1012073.s006.tif]

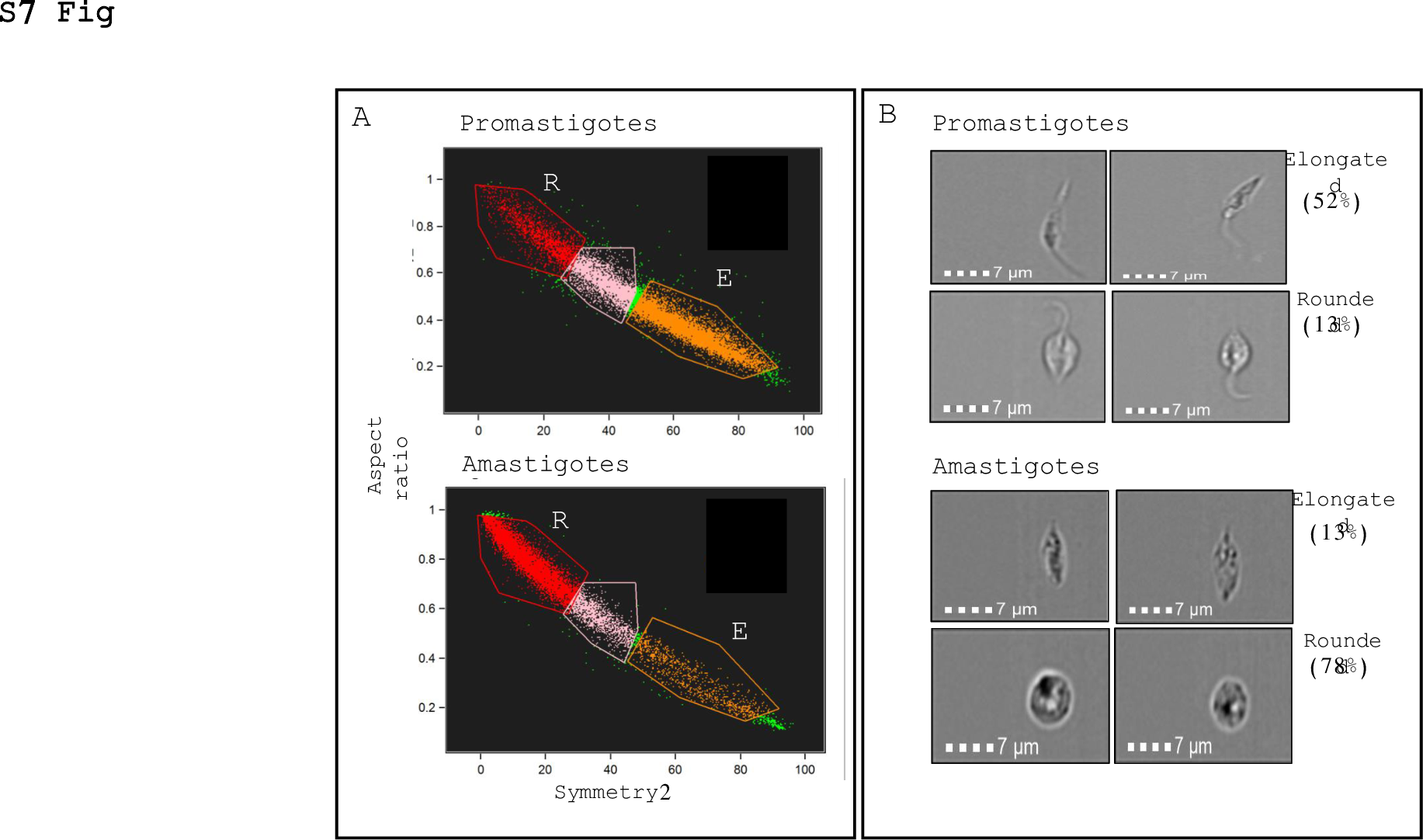

Supplement: S7 Fig — A. An Amnis Image StreamX Mk II image streamer was fed with either late-log phase axenic promastigotes (upper panel) or amastigotes (lower panel). Snapshot of 10,000 cells per each group was taken and used to calculate the width/length aspect ratio. Cells were classified based on their aspect ratio range as elongated (E, 0.1–0.4) or rounded (R, 0.6–1.0). B. Representative snapshots of elongated and rounded flagellated promastigotes (upper panel) and elongated and rounded aflagellated amastigogtes. The %age of each population is indicated. (TIF) [file ppat.1012073.s007.tif]

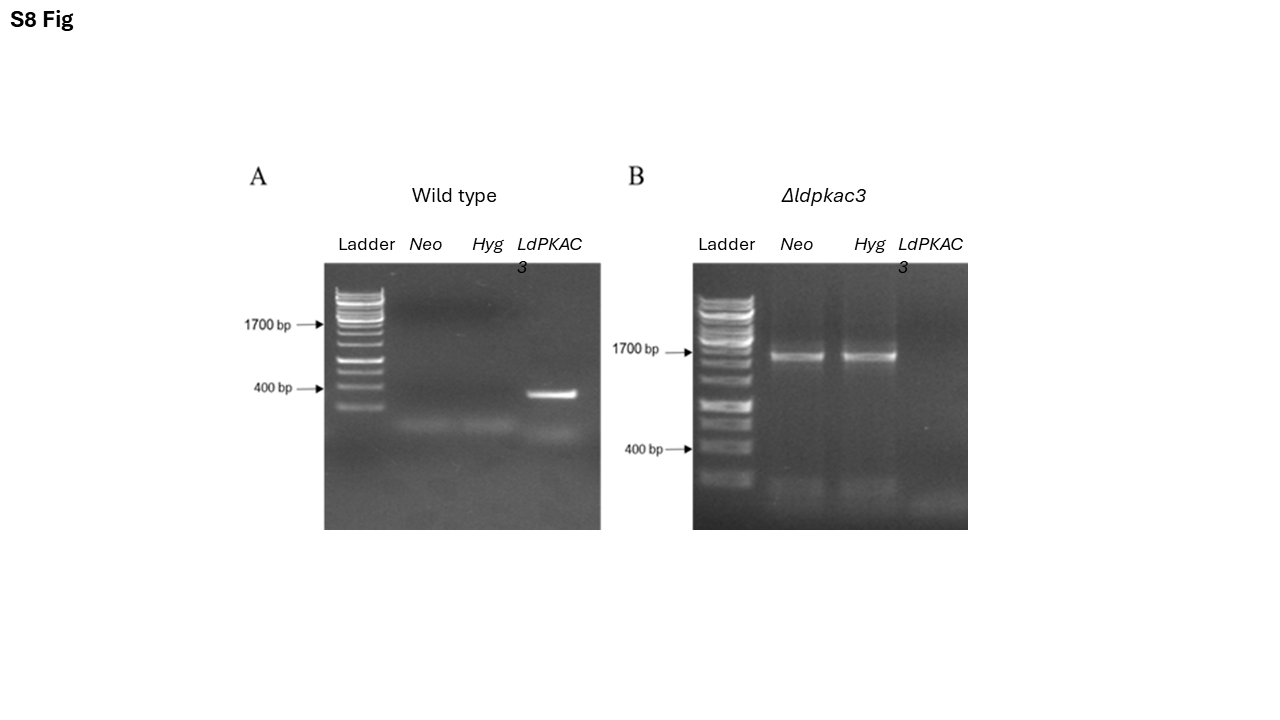

Supplement: S8 Fig — PCR on extracted genomic DNA from (A) wild type or (B) Δpkac3 cells. The PKAC3 ORF is detected only in wild type cells, while in Δpkac3 cells both resistance marker cassettes, hygromycin (Hyg) and neomycin (Neo) are amplified. (TIF) [file ppat.1012073.s008.tif]

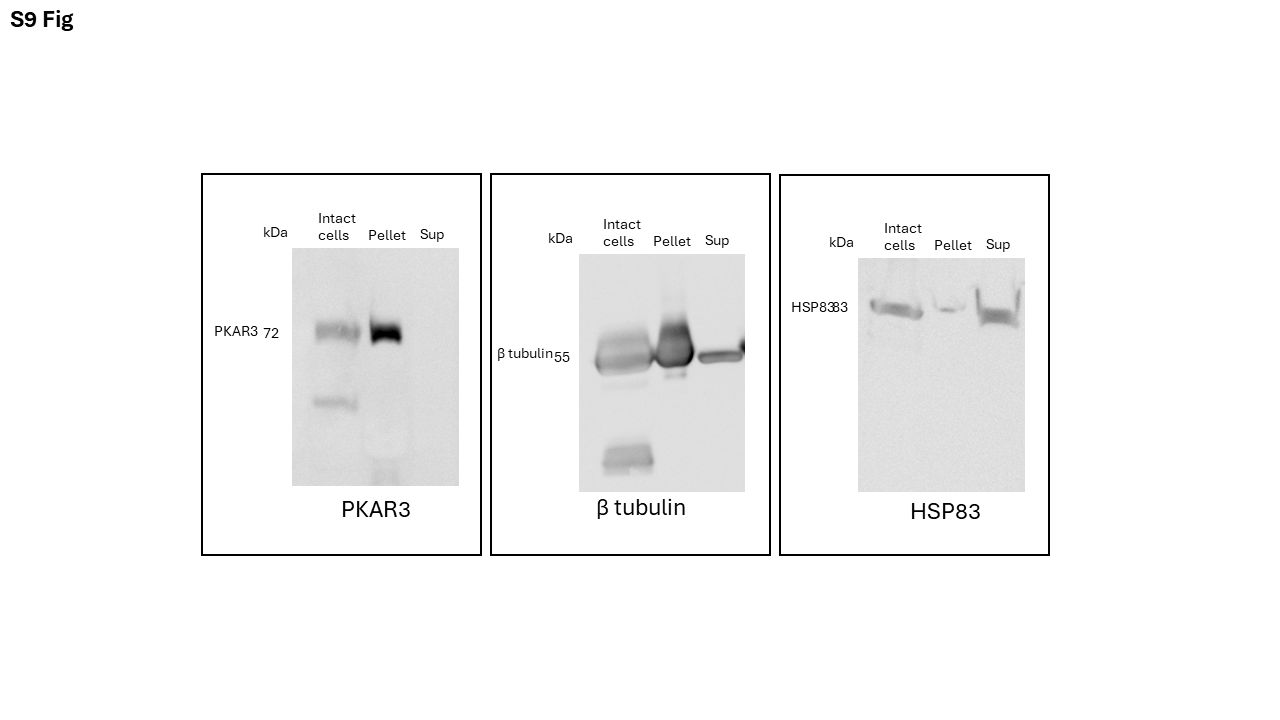

Supplement: S9 Fig — Experimental details are in the Legend to Fig 1C. Antibodies used; anti PKAR3, β tubulin and HSP83. (TIF) [file ppat.1012073.s009.tif]

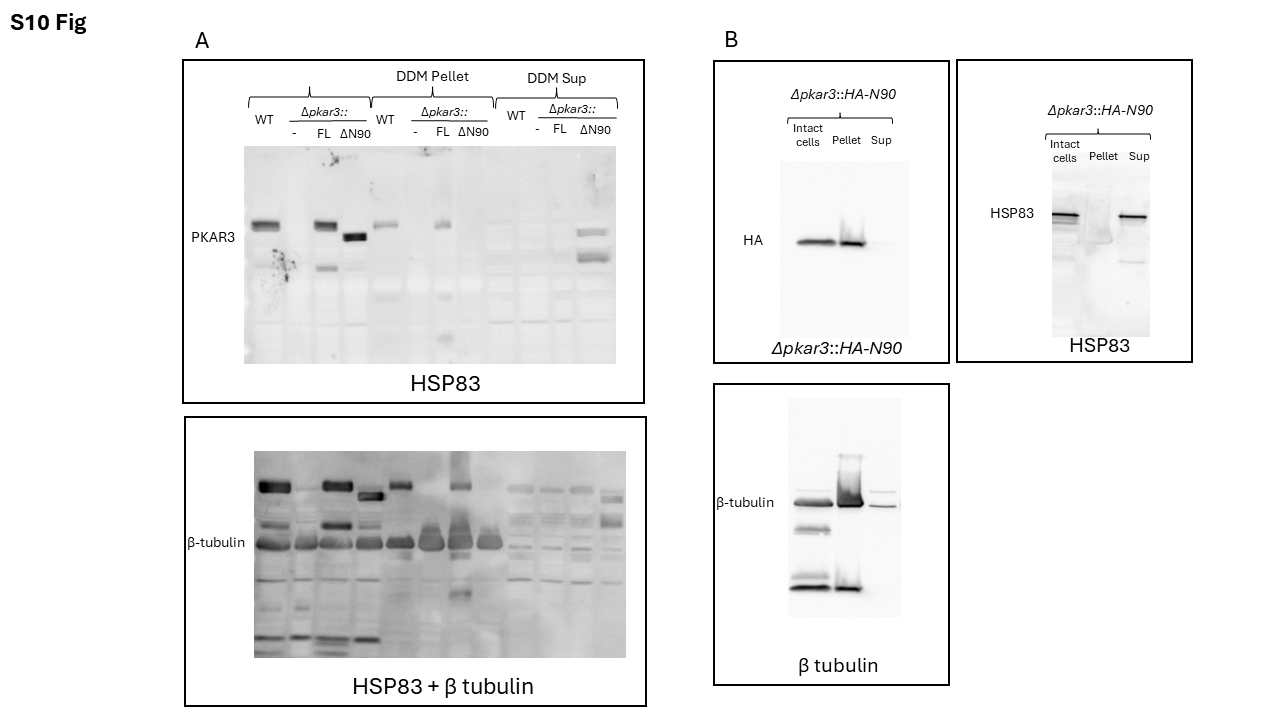

Supplement: S10 Fig — (A) the full-length gels of Fig 2A, (B) the full-length gels of Fig 2C. Experimental details are in the legends to Fig 2 (TIF) [file ppat.1012073.s010.tif]

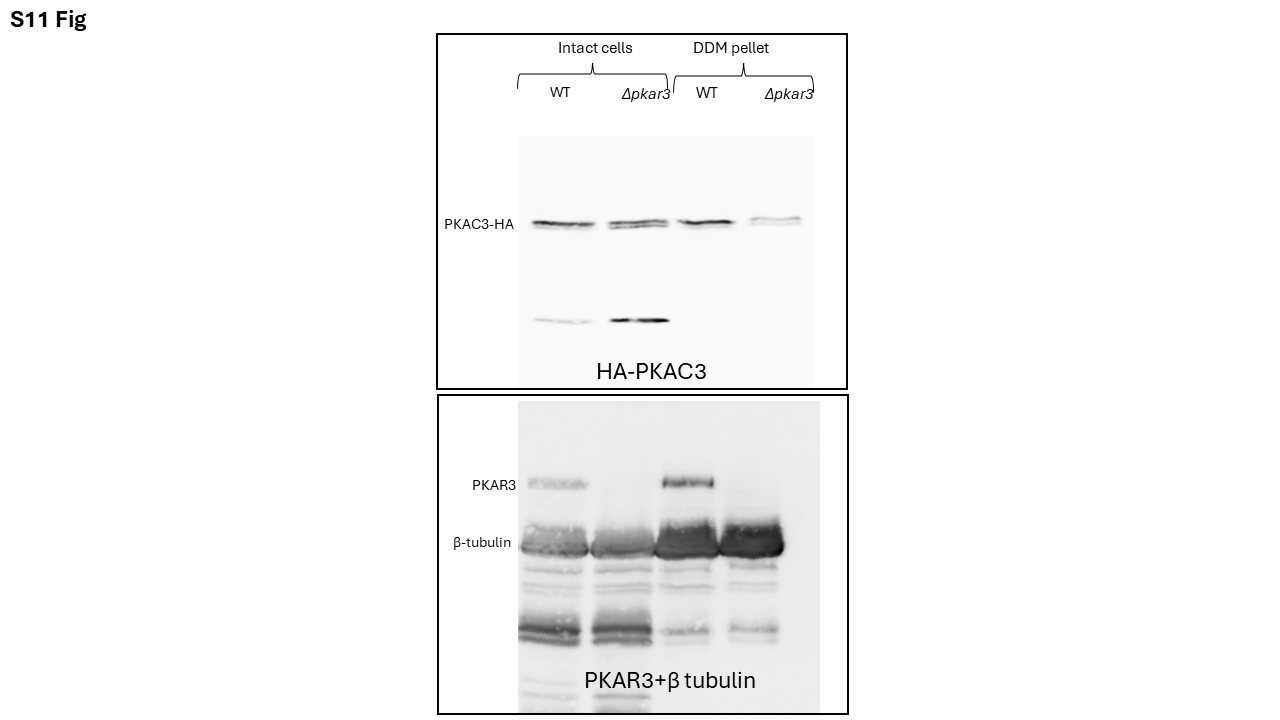

Supplement: S11 Fig — Experimental details are in the legends to Fig 5. The full-length gels of Fig 5A. (TIF) [file ppat.1012073.s011.tif]
