# Supplementary material for: A divergent protein kinase A regulatory subunit essential for morphogenesis of the human pathogen Leishmania
Source: PLoS Pathog. 2024 Mar 29;20(3):e1012073. doi: 10.1371/journal.ppat.1012073 (PMC11006142; doi:10.1371/journal.ppat.1012073)
Supplement: S1 Table — The list of PKAR genes of Kinetoplastidae in this table is from TriTrypDB website (TriTrypDB). GenBank accession numbers refer to the nucleotide sequence containing an open reading frame with a Blast hit to PKAR1 and/or PKAR3. (DOCX) [file ppat.1012073.s012.docx]

**S1 Table. A list of protein kinase A regulatory (PKAR) subunits in the *Kinetoplastidae.***

| ***Organism*** | **Host (vector)** | **Name** | **Accession number(s)** | **Source^1^** |
| --- | --- | --- | --- | --- |
| *Bodo saltans* | Free-living | Bsal_PKAR1a Bsal_PKAR1bψ Bsal_PKAR3 Bsal_PKARx Bsal_PKARy^†^ | BSAL_17215 BS32045 BSAL_8045 BSAL_35655 BSAL_19590 | TriTrypDB |
| *Paratrypanosoma confusum* | Mosquito | Pcon_PKAR1 Pcon_PKAR3ψ Pcon_PKARx | PCON_0045260 PCON_0009880 PCON_0048030 | TriTrypDB |
| *Angomonas deanei* | Assassin bug  (mosquito?) | Adea_PKAR1 Adea_PKAR3 | ADEAN_000701600 ADEAN_000115800 | TriTrypDB |
| *Blechomonas_ayalai* | Flea | Baya_PKAR1 Baya_PKAR3 | Baya_015_0760 Baya_083_0130 | TriTrypDB |
| *Crithidia fasciculata* | Mosquito | Cfas_PKAR1 Cfas_PKAR3 | CFAC1_220031000 CFAC1_290057800 | TriTrypDB |
| *Endotrypanum monterogeii*) | Sloth (Sand fly) | Emon_PKAR1 Emon_PKAR3 | EMOLV88_1300064001 EMOLV88_340030000 | TriTrypDB |
| *Herpetomonas muscarum* | Housefly | Hmus_PKAR1 Hmus_PKAR3 | AUXJ01005618 AUXJ01002838 | GenBank |
| *Leishmania donovani* | Human (sand fly) | Ldon_PKAR1 Ldon_PKAR3 | LdBPK.13.2.000160 LdBPK.34.2.002680 | TriTrypDB |
| *Leptomonas seymouri* | Cotton stainer^‡^ | Lsey_PKAR1 Lsey_PKAR3 | Lsey_0098_0190 Lsey_0369_0050 | TriTrypDB |
| *Lotmaria passim* | Honeybee | Lpas_PKAR1 Lpas_PKAR3 | AHIJ01002025 AHIJ01002162+AHIJ01001347 | GenBank |
| *Novymonas esmeraldas* | Plant bug | Nesm_PKAR1 Nesm_PKAR3 | JAECZO010000121 JAECZO010000086 | GenBank |
| *Phytomonas serpens* | Tomato (heteropteran bug) | Pser_PKAR1 | AIHY01000725 | GenBank |
| *Porcisia deanei* | Porcupine (sand fly) | Pdea_PKAR1 Pdea_PKAR3 | JAHGAS010000297 JAHGAS010000723 | GenBank |
| *Strigomonas culicis* | Mosquito | Scul_PKAR1 Scul_PKAR3 | AUXH01000039 AUXH01000346 | GenBank |
| *Trypanosoma brucei* | Livestock (tsetse fly) | Tbru_PKAR1 | Tb927.11.4610 | TriTrypDB |
| *Trypanosoma cruzi* | Human (triatomine bug) | Tcru_PKAR1 Tcru_PKAR3 | TcCLB.506227.150 TcCLB.510879.50 | TriTrypDB |
| *Trypanosomatidae sp* Fi-14 | Heteropteran bug | TspF_PKAR1 | JAKVQF01000046 | GenBank |
| *Trypanosomatidae sp* JR-2017b | Human | Tspb_PKAR1 Tspb_PKAR3a Tspb_PKAR3b | NSCP01020035 NSCP01012242 NSCP01014566 | GenBank |
| *Vickermania ingenoplastis* | Housefly | Ving_PKAR1 | VFSE01000161 | GenBank |

**^1^** GenBank accession numbers refer to the nucleotide sequence containing an open reading frame with a Blast hit to PKAR1 and/or PKAR3

ψ Pseudogene

^†^ Contains two additional pairs of cyclic nucleoside binding domains

^‡^ *Leptomonas seymouri* has a wide host range and has been isolated from humans

+ Fusion of two adjacent genes
