# Supplementary material for: A divergent protein kinase A regulatory subunit essential for morphogenesis of the human pathogen Leishmania
Source: PLoS Pathog. 2024 Mar 29;20(3):e1012073. doi: 10.1371/journal.ppat.1012073 (PMC11006142; doi:10.1371/journal.ppat.1012073)
Supplement: S2 Table — The list of PKAR genes is from Gene Databank. (DOCX) [file ppat.1012073.s013.docx]

**S2 Table. Protein kinase A regulatory (PKAR) subunits in selected other eukaryotes.**

| ***Organism*** | **Kingdom/Phylum** | **Name** | **Accession Number(s)** | **Source** |
| --- | --- | --- | --- | --- |
| *Homo sapiens* | Animalia/Chordata | Hsap_PKARIaa Hsap_PKARIb Hsap_PKARIIaa Hsap_PKARIIb | NP_001263218 NP_001158230 NP_001308911 NP_002727 | GenBank |
| *Plasmodium falciparum* | Chromista/Miozoa | Pfal_PKAR | XP_001350628 | GenBank |
| *Saccharomyces cerevisiae* | Fungi/Ascomycota | Scer_BCY1 | NP_012231 | GenBank |
| *Physcomitrella patens* | Plantae/Bryophyta | Ppat_PKAR1 Ppat_PKARx2 | XP_024360287 XP_024358631 | GenBank |
| *Naegleria gruberi* | Protozoa/Amoebozoa | (Ngru) | XP_002681660 | GenBank |
| *Giardia intestinalis* | Protozoa/Metamonad | Gint_PKAR | GL50581_1446 | GiardiaDB |
| *Trichomonas vaginalis* | Protozoa/Metamonad | Tvag_PKAR1 Tvag_PKAR2 | XP_001318981 XP_001311320 | GenBank |
| *Dictyostelium discoideum* | Protozoa/Mycetozoa | Ddis_PKAR | XP_641686 | GenBank |
