# Supplementary material for: A divergent protein kinase A regulatory subunit essential for morphogenesis of the human pathogen Leishmania
Source: PLoS Pathog. 2024 Mar 29;20(3):e1012073. doi: 10.1371/journal.ppat.1012073 (PMC11006142; doi:10.1371/journal.ppat.1012073)
Supplement: S3 Table — The list of primers and their sequence is provided. (DOCX) [file ppat.1012073.s014.docx]

**S3 Table: Primers used in this study**

| **Purpose** | **Primers** | **Fragment length** | **Restriction enzymes** | **Plasmid(s)** |
| --- | --- | --- | --- | --- |
| Expression of PKAR3 in *E. coli* | PKAR3_CDS-Fwd  PKAR3_CDS-Rev | 1976 bp | BamHI  NotI | pETDuet |
| Knockout of *PKAR3* | PKAR3 5' UTR fr  pkar3 5' UTR rev  PKAR3 5' UTR fr  PKAR3 3' UTR fr  PKAR3 3' UTR rev | 700bp  700bp  700bp  960bp  960bp | SalI  HindIII  KpnI  BamHI  XbaI | pKON  pKOH |
| Add-back of *PKAR3* | PKAR3 fr  PKAR3 rev | 1941bp | XhoI  KpnI | pNUSHnB |
| Knockout of *PKAC3* | PKAC3_5'Fwd1  PKAC3_5'Rev1 | 723 bp | KpnI  HindIII | pKON  pKOH |
|  | PKAC3_3'Fwd1  PKAC3_3'Rev1 | 1189 bp | BamHI  XbaI | pKON pKOH |
| Validation of *PKAC3* KO | PKAC3_CDS-Fwd2  PKAC3_CDS-Rev2 | 400 bp |  |  |
|  | Upstream to PKAC3 5’  Neo-Rev1  Hyg-Rev1 | 1700bp |  |  |
| Add-back of *PKAC3* | PKA_CDS-Fwd1  PKA_CDS-Rev1 | 1014 bp | NdeI  KpnI | pNUSHnB |
| Expression of PKAR3 in *T. brucei* | PKAR3_HindIII_fw  PKAR3_lr_Ty1_BamHI_rev | 1986 bp | HindIII  BamHI | pHD615 |
| Expression of PKAR1 in *L. tarentolae* | LdR Forward  LdR Reverse | 1575 bp | Xho1  Not1 | pLEXY_I-ble3 |
| Expression of PKAR3 in *L. tarentolae* | LdR' Forward  LdR' Reverse | 2010 bp | Xho1  Not1 | pLEXY_I-ble3 |
| Expression of PKAC3 in *L. tarentolae* | GG_3 LdC3 forward  GG_4 LdC3 reverse | 1035 bp | NcoI  NotI | pLEXY_I-neo |
| Expression of  PKAR3(321-647) in  *E.coli* | PKAR3(321-647)_FW  PKAR3(321-647)_RV | 1232 bp | BamHI  NotI | pETSUMO |

**Primer sequences**

Upper case letters denote sequence that matches the target, while lower case letter represent sequence added to incorporate restriction sites (underlined) and epitope tags.

PKAR3_CDS-Fwd: gccaggatccggaaaacctgtattttcagggatctATGTCCAGTTTCGAC

PKAR3_CDS-Rev: attatgcggccgcCTACGCGCTCTCGTG

PKAC3_5'Fwd1: gtaccggtaccTGCGCCGCGACAGAGACAG

PKAC3_5'Rev1: ctggtaagctTGGTGTTCTATTATGACTGGTAAGG

PKAC3_3'Fwd1: gactgggatccTATCCCATTTCTGCCTCCAAGG

PKAC3_3'Rev1: ctgactctagaGAGAATGTGCGTTGATGTGG

PKAC3_CDS-Fwd2: GGCCGAAGCTTCCAAGTGG

PKAC3_CDS-Rev2: GCGGTAAACGATCGTCTTG

PKAR3_HindIII_fw: gcgacaagcttATGTCCAGTTTCGAC

PKAR3_lr_Ty1_BamHI_rev: gcgacggatcctcagtcaagtgggtcctggttagtatggacctcagatccCGCGCTCTCGTGTTG

PKA_CDS-Fwd1: ggctacatatgtacccatacgacgtcccagactacgctATGATCACCAAGGCCGAAG

PKA_CDS-Rev1: ctgacggtaccCTACTCGTCCGTATACTGGCC

PKAR3 5' UTR fr: aatctGTCGACggcatcattcgtaattcc

pkar3 5' UTR rev: aatctAAGCTTaaagggcaaggcgacagg

PKAR3 5' UTR fr: aatctGGTACCcattcgtaattcctctcc

PKAR3 3' UTR fr : aatctGGATCCaagccacagctttctacc

PKAR3 3' UTR rev: aatctTCTAGAacatgtaaaagcatcgccc

PKAR3 fr : AATCACTCGAGTCCAGTTTCGACAAATACG

PKAR3 rev: AATCAGGTACCCTACGCGCTCTCGTGTTG

Hyg-Rev1: AATGTCAAGCACTTCCGG

Neo-Rev1: CAGCTGCGCAAGGAACGC

Upstream to PKAC3 5’: GGTGGCTGGTGGCTGGCAG

Primer R1 fw: ccgcctcgagatgggcagcagccatcaccatcatcaccacagccaggatccggaaaacct

gtattttcagggatctATGTCCGCGGAAGACACCCCC

Primer R1 rv: gagggcggccgccttaCTGGACCGCCGCCGGGGCACC

Primer C3 fw: gccaccagatctgccatggcttcggcttggagccacccgcagttcgaaaaagctATCACC

AAGGCCGAAGCTTCC

Primer C3 rv: aggagggcggccgcCTACTCGTCCGTATACTGGCCGTT

Primer R3 fw: ccgcctcgagatgggcagcagccatcaccatcatcaccacagccaggatccggaaaacctgtatttt

cagggatctATGTCCAGTTTCGACAAATAC

Primer R3 rv: gagggcggccgcCTACGCGCTCTCGTGTTGTCGCAG

PKAR3(321-647)_FW: TAG TGG ATC CCA GCG CCC AAG CCG CGG

PKAR3(321-647)_RV: att atg cgg ccG CTT ACT GGA CCG CCG C
